# Electrical Control of the Nuclear Spin States of Rare-Earth Adatoms

**DOI:** 10.1021/acsnano.4c16416

**Published:** 2025-04-21

**Authors:** Homa Karimi, Aleksander L. Wysocki, Kyungwha Park

**Affiliations:** † Department of Physics, Virginia Tech, Blacksburg, Virginia 24061, United States; ‡ Department of Physics and Astronomy, University of Nebraska at Kearney, Kearney, Nebraska 68849, United States

**Keywords:** rare-earth adatoms, nuclear spin qubits, multiconfigurational ab initio, hyperfine coupling, electrical control

## Abstract

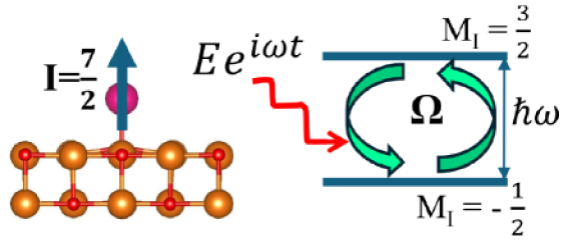

Rare-earth adatoms on surfaces have been studied for potential atomic-scale magnetic storage, quantum sensing, and quantum computing applications. Despite accumulating experimental efforts, a comprehensive description of the electronic configurations of the adatoms remains elusive. Here, we investigate two charge states and several electronic configurations, including 5d and 6s valence shells, for a Sm adatom on a MgO substrate using multiconfigurational *ab initio* methods, for the possibility of using the Sm nuclear spin levels as qubits. For the configurations in a neutral charge state, we find that the electronic ground state is a singlet, and thus the hyperfine interaction associated with the ^147^Sm nucleus is absent, which may greatly enhance nuclear spin coherence time. The degeneracy of the nuclear levels is lifted by the nuclear quadrupole interaction. We show that the splitting of the nuclear levels can be controlled by a static electric field, and that Rabi oscillations between the nuclear levels can be induced by a time-dependent electric field. For the configurations in a singly charged state, electronic Kramers doublets are formed. The electronic configurations including an unpaired 6s orbital exhibit a strong hyperfine Stark effect due to a large Fermi contact contribution to the hyperfine interaction. In these configurations, electric-field-induced Rabi oscillations between the electronic-nuclear levels can occur at frequencies up to 3 orders of magnitude higher than those for the neutral charge state. The proposed system may be experimentally observed within scanning tunneling microscopy.

There have been great efforts to experimentally investigate and manipulate magnetic properties of 3d transition-metal or 4f rare-earth adatoms on substrates.^1−12^ Recently, electron spin resonance (ESR) technique has been combined with a scanning tunneling microscopy (STM),^13,14^ which facilitated direct probing of the electronic levels and electronic-nuclear levels of magnetic adatoms and their utilization for quantum computation. Magnetic adatoms can be coupled via exchange and dipolar interactions on substrates. The quantum states of the coupled adatoms were experimentally shown to be initialized, detected, and coherently controlled.^12^ Single-qubit and multiple-qubit operations were also demonstrated using coupled magnetic adatoms.^12^

One of the desirable properties of spin qubit systems is a long spin coherence time which can be hampered by various interactions with the environment. In the ESR-STM setup, the electron spin qubits based on 3d transition-metal adatoms showed a rather short spin coherence time^12^ compared to other spin qubit systems. In order to increase a spin coherence time, 4f rare-earth adatoms were proposed to be utilized because 4f orbitals are strongly localized and appear well below the Fermi level.^10,11^

One route to further enhance the spin coherence time is to utilize pure nuclear spin levels as qubits without the interaction between the electronic magnetic moment and the nuclear spin moment, i.e., hyperfine interaction. Recently, the nuclear spin state of an Eu(III)-based molecule (that bears zero total angular momentum *J* = 0 in the ground state) was shown to be optically initialized and coherently controlled with an exceptionally narrow line width and a long spin coherence time^15^ compared to molecules based on 3d elements or other 4f elements. In addition, the nuclear spin levels of a single ^123^Sb^+^ donor (that has zero spin *S* = 0) in silicon was shown to have several orders of magnitude longer spin coherence time than the systems where the hyperfine interaction is present.^16^ So far, adatom systems analogous to these two examples have not been discussed.

Regarding control of electronic spin or nuclear spin levels for quantum information science applications, a magnetic field is typically applied. However, a magnetic field cannot be confined locally. In order to address individual qubits locally, operations and control of the spin levels using an electric field would be beneficial. The nuclear spin levels of a single ^31^P donor or a single ^123^Sb^+^ donor in silicon were shown to be electrically controlled to exhibit hyperfine Stark effect or Rabi oscillations.^16,17^ Similarly, the electronic-nuclear spin levels of Tb(III)-based molecules were also shown to be electrically controlled within a single molecule transistor setup.^18^

In order to use magnetic adatoms as quantum sensors or atomic qubits, it is essential to understand the electronic and magnetic properties of them. The first step toward such an understanding is to identify the electronic configurations of the adatoms. This task is typically carried out by X-ray absorption spectroscopy (XAS) or X-ray magnetic circular dichroism (XMCD).^6,8,10,19^ In these experiments, the occupancy of the 4f orbitals was routinely determined. Although it is rare, the occupancies of 6s and 5d valence orbitals and the resultant charge state can be identified from XAS and XMCD combined with multiplet calculations.^8^ In addition, depending on the type of adatom and substrate and substrate thickness, different electronic configurations can coexist.^6^

Here we consider two charge states and several electronic configurations of a samarium (Sm) adatom on a MgO substrate for two adsorption sites. We investigate their magnetic properties by using quantum chemistry methods. Our study can be complementary to XAS and XMCD experiments. Interestingly, in a neutral Sm case, the electronic ground state is a singlet and so the hyperfine interaction for the Sm nucleus is absent, which bolsters a long spin coherence time of the Sm nuclear spin states. We show a significant quadrupole Stark effect and compute Rabi oscillations between the nuclear levels induced by a time-dependent electric field. In a singly charged Sm case, we find a large hyperfine Stark effect and electric-field induced Rabi oscillations between electronic-nuclear levels with high Rabi frequencies.

## Results and Discussion

**Figure 1:**
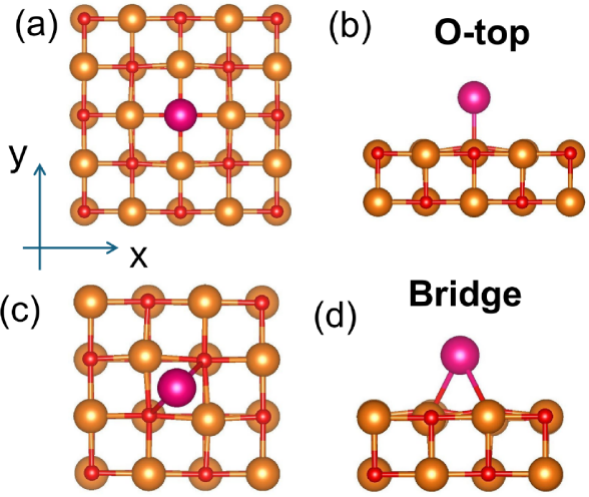
(a) Top view and (b) side view of a Sm adatom on top of an oxygen atom (O-top site) at MgO(001) surface. (c) Top view and (d) side view of a Sm adatom above the midpoint between two nearest neighboring oxygen atoms (bridge site) at MgO(001) surface. The color code is as follows: Mg (orange), O (red), Sm (magenta). The *z* axis is normal to the MgO surface.

A magnetic adatom on MgO can be adsorbed at three adsorption sites: (i) on top of an O atom (i.e., O-top site); (ii) above the midpoint between two nearest neighboring O atoms (i.e., bridge site) (see Figure 1); (iii) on top of a Mg atom (i.e., Mg-top site) at the MgO(001) surface. The density-functional theory (DFT) binding energies of the O-top, bridge, and Mg-top sites are 2.74, 2.29, and 0.77 eV, respectively. Therefore, our DFT calculations show that the O-top site is the most stable. It should be noted, however, that for kinetic reasons (or for different environments) other adsorption sites can be realized experimentally. For example, for some rare-earth adatoms on MgO, both O-top and bridge sites have been observed.^6,10^ For this reason, we also investigate the bridge site. Recently, for Sm adatoms on MgO, the Mg-site has also been observed. However, the experimental dI/dV spectrum for the Mg-site was featureless,^20^ and so we do not consider the Mg-top site in our study.

Our DFT calculations indicate only a tiny charge transfer between the Sm adatom and the surface, which corresponds to a neutral Sm oxidation state. However, for experimental samples, the oxidation state of adatoms on surfaces is often affected by extrinsic factors (defects, gate voltage, thickness of MgO layers above a metallic substrate etc.) which are not included in our calculations. We, therefore, consider neutral and singly charged oxidation states in our quantum chemistry calculations. We also examine several electronic configurations for each oxidation state at each adsorption site since it was experimentally demonstrated that for some rare-earth adatoms, different electronic configurations can coexist^6,10^ and the dominant configuration depends on the thickness of MgO layers overlaid on an Ag substrate.^6^ Individual electronic configurations have unique properties. Our results will be useful for identification of the electronic configurations along with XAS and XMCD experimental techniques.

The relaxed structures of the two adsorption sites are obtained from DFT. The calculation details can be found in the Methods section. For each adsorption site, considering two charge states (Sm^0^ and Sm^1+^) and a few electronic configurations, we perform multiconfigurational *ab initio* calculations of the electronic structure and determine the nuclear spin and electronic-nuclear spin states for a ^147^Sm nucleus (14.99% natural abundance). (Sm has two isotopes with nonzero nuclear spin. Here we focus on the ^147^Sm nucleus, but the results for the other isotope can be found by appropriately rescaling our data.) We use the complete active space self-consistent field (CASSCF) method combined with the restricted active space state interaction (RASSI)^21^ for the treatment of spin–orbit coupling. The calculation details are described in the Methods section.

### Sm^0^ Case

A neutral Sm atom in the gas phase has an electronic configuration 4f^6^5d^0^6s^2^ in the ground state. Hund’s rule dictates that the orbital angular momentum *L* = 3 and the spin angular momentum *S* = 3, giving rise to the total angular momentum *J* = 0 in the electronic ground state. For a Sm^0^ adatom on MgO, we investigate the following two electronic configurations: (i) 4f^6^5d^0^6s^2^; (ii) 4f^5^5d^1^6s^2^. The first configuration is the same as that of a neutral Sm atom in the gas phase, whereas in the second configuration there are five electrons in 4f orbitals and one 5d orbital is occupied. The configurations, 4f^*n*^5d^0^6s^2^ and 4f^*n*–1^5d^1^6s^2^, were studied for other rare-earth elements on metallic substrates,^19,22^ where *n* is the number of 4*f* electrons for a neutral rare-earth atom. For the *ab initio* calculations of the two electronic configurations, we construct the following active spaces. For the first electronic configuration, we consider an active space consisting of six electrons and seven 4f orbitals, which is referred to as CAS6-7. For the second electronic configuration, an active space comprises six electrons and 12 orbitals such as seven 4f orbitals and five 5d orbitals which is referred to as CAS6-12. In both configurations, a 6*s* orbital is excluded in the active space because it is confirmed to be doubly occupied even when it is included in the active space. We investigate each configuration separately.

For the configuration 4f^6^5d^0^6s^2^, the electronic ground state is a singlet. The ground singlet is well separated from the first excited quasi-doublet or state, similarly to a neutral Sm atom in the gas phase. For the O-top site, the electronic ground-state energy is separated from the first excited quasi-doublet by 159 cm^–1^, while for the bridge site, it is separated from the first excited state by 65 cm^–1^. These different energy separations for the O-top and bridge sites arise from different crystal fields. Figure S1 and Table S1 shows electronic excitation energies for the O-top and bridge sites compared to those for an isolated neutral Sm atom. As shown in Figure S1 and Table S1, the excitation energies for the O-top and bridge sites are quite different from those for an isolated neutral Sm atom. For the Sm adatom on MgO, the crystal field is comparable to the spin–orbit coupling, and so the excited electronic levels cannot be categorized by multiplets with effective *J* values like in the isolated atom case. This trend is applied to most of the electronic configurations that we consider in the current work. Furthermore, the hyperfine interaction for the electronic ground singlet is approximated to be unobservable even when we consider the effect of the excited electronic states because the hyperfine coupling is at least several orders of magnitude smaller than the spin–orbit coupling and crystal field. The separation between the electronic first-excited state and ground state is several orders of magnitude larger than the energy of the interaction between the quadrupole moment of the ^147^Sm nucleus (nuclear spin *I* = 7/2) and an electric field gradient at the nucleus, i.e., nuclear quadrupole interaction. Therefore, the interaction parameters can be calculated using the ground singlet wave function. The nuclear quadrupole interaction Hamiltonian can be written as^23^
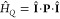
1
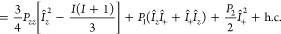
2where *P*_1_ = *P*_*xz*_ – i*P*_*yz*_ and . **P** is the nuclear quadrupole tensor and **Î** is the nuclear spin angular momentum operator. The nuclear quadrupole tensor elements are calculated by following the method discussed in ref (23).

**Figure 2:**
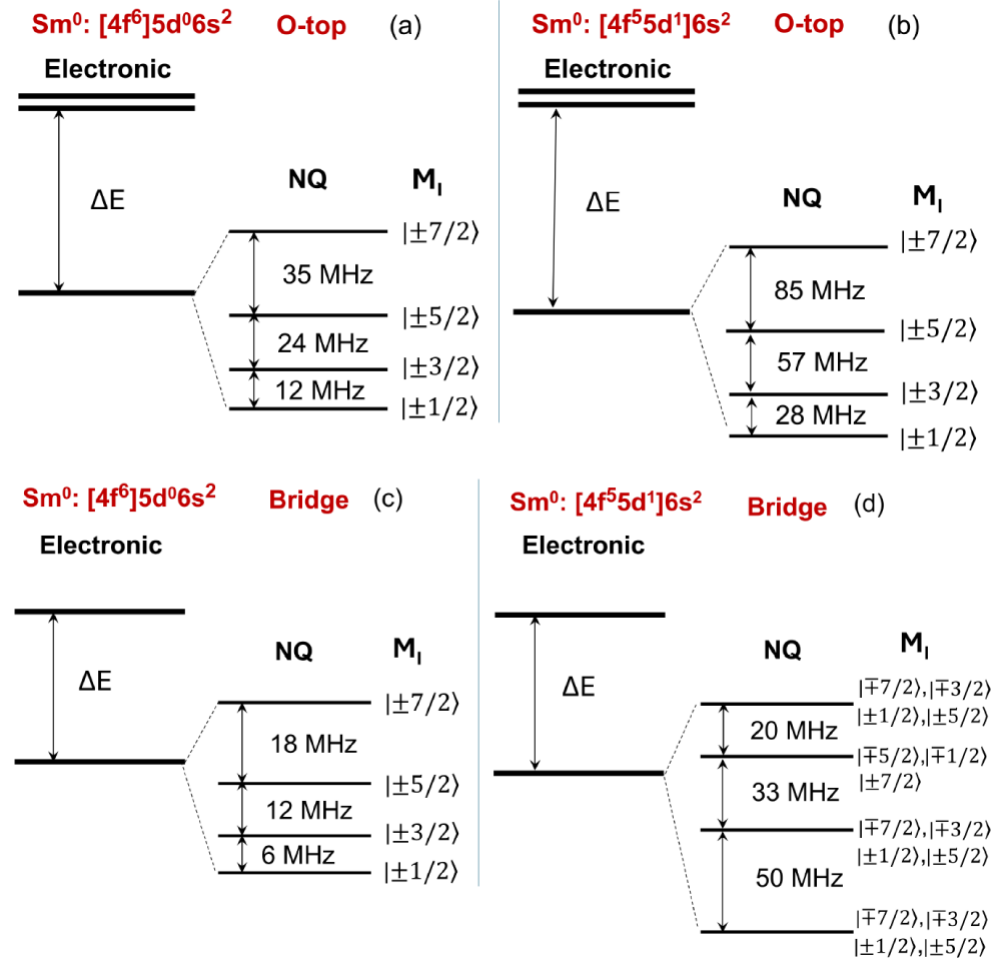
Schematic diagrams of the low-energy nuclear levels split by the nuclear quadrupole interaction for the ^147^Sm nucleus (a), (b) for the O-top site and (c), (d) for the bridge site in the electronic configurations 4f^6^5d^0^6s^2^ and 4f^5^5d^1^6s^2^, respectively. For the bridge site, only dominant *M*_*I*_ states are shown. In (a)–(d), each nuclear level is doubly degenerate.

Table 1 lists our calculated nuclear quadrupole tensor for the ^147^Sm nucleus for the configuration 4f^6^5d^0^6s^2^ at the O-top and bridge sites. Since the O-top site has *C*_4*v*_ point group symmetry, the nuclear quadrupole tensor becomes a diagonal matrix with *P*_*xx*_ = *P*_*yy*_. Thus, only the *P*_*zz*_ term survives in eq 2. This leads to a splitting of the nuclear spin levels for the electronic ground state into four doubly degenerate levels, *M*_I_ = ± 1/2, ± 3/2, ± 5/2, and ± 7/2, where *M*_I_ is the eigenvalue of the *z* component of the nuclear spin angular momentum operator *Î*_*z*_. (Here the *z* axis is normal to the MgO surface.) Since the *P*_*zz*_ value is positive, the *M*_I_ = ± 1/2 levels are the nuclear ground doublet, and the separation between the neighboring nuclear levels increases as the energy increases (see Figure 2a). The energies of these separations are about several tens of MHz. For the bridge site, since it has *C*_2*v*_ point group symmetry, we find that *P*_*xz*_ = *P*_*yz*_ = 0 but *P*_*xy*_ ≠ 0, which results in *P*_1_ = 0 and *P*_2_ ≠ 0. The electronic ground state is again split into four doubly degenerate nuclear levels which are not pure *M*_*I*_ states due to weak mixing between nuclear levels differing by Δ*M*_I_ = ± 2. The separation between the neighboring nuclear levels is about half of that for the O-top site due to the decrease in *P*_*zz*_ (see Figure 2c).

**Table 1:** Calculated Elements of the Nuclear Quadrupole Tensor in MHz for ^147^Sm^0^ at the O-Top and Bridge Sites for Active Space CAS6-7 and CAS6–12 (Or for Electronic Configurations 4f^6^5d^0^6s^2^ and 4f^5^5d^1^6s^2^)^a^

Site	Configuration	CASSCF	*P*_*xx*_	*P*_*xy*_	*P*_*xz*_	*P*_*yy*_	*P*_*yz*_	*P*_*zz*_
O-Top	[4f^6^]5d^0^6s^2^	CAS6-7	–1.96	0.00	0.00	–1.96	0.00	3.92
O-Top	[4f^5^5d^1^]6s^2^	CAS6-12	–4.71	0.00	0.00	–4.71	0.00	9.42
Bridge	[4f^6^]5d^0^6s^2^	CAS6-7	–0.98	0.08	0.00	–0.98	0.00	1.96
Bridge	[4f^5^5d^1^]6s^2^	CAS6-12	–1.08	4.56	0.00	–1.08	0.00	2.16

a Active orbitals are within brackets.

Let us now discuss changes of the nuclear quadrupole tensor for the ^147^Sm nucleus in the presence of a static electric field applied along the *z* axis for the configuration 4f^6^5d^0^6s^2^ (Table 2). The changes of the nuclear quadrupole tensor, Δ*P*_*xx*,*yy*_/*P*_*xx*,*yy*_, are about a few percents with an electric field of 1 GV/m which can be typically realized within a STM setup. This quadrupole Stark effect is more significant for the bridge site than for the O-top site due to the low symmetry of the bridge site.

**Table 2:** Calculated Elements of the Nuclear Quadrupole Tensor in MHz for ^147^Sm at the O-Top and Bridge Sites for Active Space CAS6-7 and CAS6-12 in the Presence of an Electric Field of 0.002 A.U. (≈1 GV/M) Along the *z* Axis

Site	Configuration	CASSCF	*P*_*xx*_	*P*_*xy*_	*P*_*xz*_	*P*_*yy*_	*P*_*yz*_	*P*_*zz*_
O-Top	[4f^6^]5d^0^6s^2^	CAS6-7	–2.01	0.00	0.00	–2.01	0.00	4.02
O-Top	[4f^5^5d^1^]6s^2^	CAS6-12	–4.75	0.00	0.00	–4.75	0.00	9.50
Bridge	[4f^6^]5d^0^6s^2^	CAS6-7	–1.05	0.05	0.00	–1.05	0.00	2.10
Bridge	[4f^5^5d^1^]6s^2^	CAS6-12	–1.15	4.71	0.00	–1.15	0.00	2.30

**Figure 3:**
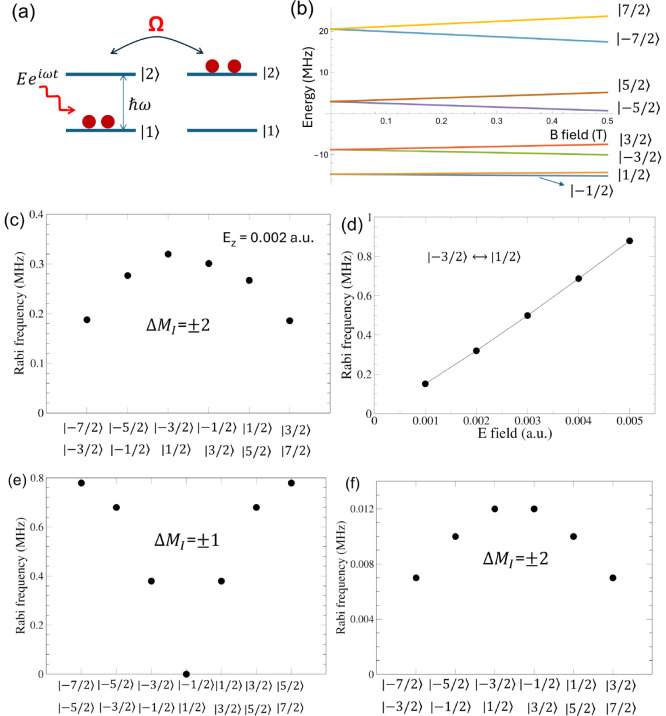
(a) Schematic of Rabi oscillations between two eigenstates |1⟩ and |2⟩ of the unperturbed system induced by a time-dependent electric field *E*e^iω*t*^. (b) Zeeman diagram of the nuclear levels and (c) Rabi frequencies for the Sm^0^ configuration 4f^6^5d^0^6s^2^ at the bridge site. (d) Rabi frequency for the transition between the *M*_I_ = −3/2 and *M*_I_ = 1/2 levels as a function of an electric field along the *z* axis for the bridge site. (e), (f) Rabi frequencies for the O-top site when an electric field whose amplitude is 0.002 au is applied along the *x* axis. In (c), (e), and (f), nuclear levels involved with Rabi oscillations are listed.

Next we examine an effect of a time-dependent electric field **E**e^iω*t*^ on the system. The electric field modifies the nuclear quadrupole tensor. The change of the nuclear quadrupole interaction Hamiltonian referred to as δ*Ĥ*_*Q*_ can be considered as a perturbation. When the angular frequency ω of the electric field multiplied by *ℏ* coincides with the energy difference between nuclear levels |*i*⟩ and |*j*⟩ (of the unperturbed system), the population of the two nuclear levels oscillates with the Rabi frequency Ω = ⟨*j*|δ*Ĥ*_*Q*_|*i*⟩/*ℏ*, according to perturbation theory. Figure 3a illustrates schematically Rabi oscillations. In order to realize Rabi oscillations in experiments, a small magnetic field is typically applied to break the degeneracy of the doublets and vary the energy difference between the nuclear spin levels such that it can resonate with the frequency of an external electric field. Figure 3(b) shows the Zeeman diagram for the electronic configuration 4f^6^5d^0^6s^2^ for the bridge site when the nuclear Zeeman Hamiltonian  is applied in addition to the nuclear quadrupole interaction Hamiltonian, where the nuclear *g* factor, *g*_N_, is −0.2328, and μ_N_ and *B*_*z*_ are the nuclear magneton and the *z* component of the magnetic field, respectively. For the bridge site, a time-dependent electric field along the *z* axis can induce Rabi oscillations between two nuclear levels whose quantum numbers differ by ±2 (Δ*M*_*I*_ = ± 2) because δ*Ĥ*_*Q*_ includes the nonzero *P*_*xy*_ (or *P*_2_) value. These oscillations can be experimentally realized by nuclear electric resonance (NER) spectroscopy.^16,24^ We compute Rabi frequencies of six transitions for the bridge site at a magnetic field 0.5 T applied along the *z* axis (Figure 3c). The Rabi frequencies increase linearly as the electric field increases, as shown in Figure 3d for the transition between the levels |*M*_I_ = −3/2⟩ and |*M*_I_ = 1/2⟩. For the O-top site, the *P*_1_ and *P*_2_ terms in δ*Ĥ*_*Q*_ due to an electric field along the *z* axis are zero by symmetry and, therefore, Rabi oscillations can occur when a time-dependent electric field is applied away from the *z* axis. Figure 3e,f shows calculated Rabi frequencies for the O-top site when a time-dependent electric field is applied along the *x* axis in the presence of a magnetic field 0.5 T applied along the *z* axis. The electric field along the *x* axis can modulate the quadrupole interaction such that *P*_*xz*_ ≠ 0 and *P*_*xx*_ ≠ *P*_*yy*_, i.e., the *P*_1_ and *P*_2_ terms in δ*Ĥ*_*Q*_ are not zero, which allows Rabi oscillations between two |*M*_*I*_⟩ levels whose quantum numbers differ by Δ*M*_I_ = ± 1 or Δ*M*_I_ = ±2.

For the electronic configuration 4f^5^5d^1^6s^2^, our calculations reveal that the electronic ground state is also a singlet with a large separation from the first excited electronic quasi-doublet or state. (For the O-top site, the separation is about 24 cm^–1^, while for the bridge site, it is about 46 cm^–1^.) This electronic ground singlet is neither the *J* = 0 state nor the *M*_*j*_ = 0 state. As shown in Table S2, it is difficult to assign an effective *J* value to the low-lying electronic states because the spin–orbit coupling is comparable to the crystal field. The hyperfine coupling for the electronic ground state can be approximated to be zero due to the same reason discussed for the configuration 4f^6^5d^0^6s^2^, and the nuclear spin levels for the electronic ground state are split by the quadrupole interaction into four Kramers doublets. Using the calculated quadrupole coupling parameters (Table 1) we find that for the O-top site, the separations between the neighboring nuclear spin levels are somewhat larger than those for the configuration 4f^6^5d^0^6s^2^ (see Figure 2b) due to the increase in *P*_*zz*_. For the bridge site, interestingly, *P*_*xy*_ is greater than *P*_*zz*_, which allows strong mixing between *M*_*I*_ levels that differ by Δ*M*_*I*_ = ±2 (see Figure 2d). (The ground state has a significant contribution from 5d_*xy*_ and  orbitals, which gives rise to a significant contribution to *P*_*xy*_.) As a result, the nuclear doublets cannot be characterized by the magnitude of *M*_I_. In addition, the separation between the neighboring nuclear spin levels is the largest for the nuclear ground doublet and the first excited doublet, and it decreases as the energy increases.

Our calculations show that the effect of a static electric field on the nuclear quadrupole tensor for the configuration 4f^5^5d^1^6s^2^ is similar to that for the configuration 4f^6^5d^0^6s^2^ (see Table 2). For the bridge site in the configuration 4f^5^5d^1^6s^2^, Rabi oscillations between the nuclear spin levels can occur with frequencies of up to an order of 0.1 MHz when a time-dependent electric field whose amplitude is 0.002 au is applied along the *z* axis in the presence of an external magnetic field (see Tables S3 and S4 for the details). For the O-top site, Rabi oscillations can be induced by a time-dependent electric field applied along any direction but the *z* axis. For example, when a time-dependent electric field is applied along the *x* axis, Rabi oscillations associated with Δ*M*_I_ = ± 1 have frequencies of an order of 1 MHz, while those associated with Δ*M*_I_ = ±2 have frequencies of an order of 0.01 MHz (see Figure S2 for the details)

### Sm^1+^ Case

Now we present our results for the Sm^1+^ adatom on MgO. In this case, we examine the following three electronic configurations: (i) 4f^5^5d^0^6s^2^; (ii) 4f^6^5d^0^6s^1^; (iii) 4f^5^5d^1^6s^1^. The three configurations, 4f^*n*–1^5d^0^6s^2^, 4f^*n*^5d^0^6s^1^, and 4f^*n*–1^5d^1^6s^1^, were studied for other rare-earth adatoms on different substrates.^2,5,6^ For the first configuration, we consider an active space consisting of five electrons and seven 4f orbitals which is referred to as CAS5-7, while for the second configuration, we choose an active space comprising seven electrons and eight orbitals (i.e., seven 4f orbital and one 6s orbital) referred to as CAS7-8. In the CAS7-8 calculation, we confirm that the 6*s* orbital is hybridized with the  orbital, although the 6s orbital contribution is dominant over the 5d orbital contribution. In order to emphasize this hybridization, the electronic configuration for CAS7-8 can be also written as 4f^6^(6s5d)^1^, and the importance of the hybridization on the intra-atomic exchange energy was discussed in ref (5). In the third configuration, the active space consists of seven electrons and 13 orbitals (seven 4f, five 5d, and one 6s orbitals) referred to as CAS7-13. For all three configurations, the total number of valence electrons is odd and so Kramers doublets are expected. We investigate each configuration separately.

For the configuration 4f^5^5d^0^6s^2^ (CAS5-7), Hund’s rule dictates that *L* = 5 and *S* = 5/2, giving rise to *J* = 5/2 in the electronic ground state. For the O-top and bridge sites, our CAS5-7 calculation shows that the six lowest electronic energy states seem to have in-plane magnetic anisotropy. This result appears to be consistent with the fact that the prolate electron density of Sm 4f^5^ favors in-plane magnetic anisotropy.^25^ However, as discussed earlier in the Sm^0^ case, especially for the O-top site, since the crystal field is comparable to the spin–orbit coupling (as shown in Figure S3 and Table S5), the six lowest states are not well separated from the higher-energy states and thus an effective spin *J* = 5/2 cannot be assigned to the O-top site. For the bridge site, we can assign *J* = 5/2 to the electronic ground multiplet (see Figure S3) and calculated crystal-field parameters are listed in Table S6. For the O-top site, the electronic first-excited Kramers doublet is separated from the ground Kramers doublet by 225 cm^–1^. For the bridge site, the ground Kramers doublet is separated from the first excited Kramers doublet by 174 cm^–1^. For both sites, the energy gap between the ground Kramers doublet and the first excited Kramers doublet is several order of magnitude larger than the energy scales of the hyperfine and nuclear quadrupole interactions. Therefore, the parameters of the hyperfine and quadrupole interactions can be, thus, calculated within the electronic ground doublet subspace.

The effective pseudospin Hamiltonian for the hyperfine interaction can be written as
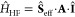
3
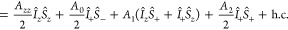
4where , *A*_1_ = *A*_*xz*_ – *iA*_*yz*_, and  Here **A** is the hyperfine coupling tensor and **Ŝ**_eff_ is the effective electronic spin angular momentum operator. For the electronic ground Kramers doublet, *S*_eff_ = 1/2. The hyperfine interaction consists of three components:^23,26^ (i) Fermi contact term which describes the interaction between the electron spin density at the nuclear position and the nuclear spin moment; (ii) spin-dipole term which is the interaction between the nuclear spin moment and the electronic spin moment; (iii) paramagnetic spin–orbital term which represents the interaction between the one-electron orbital angular momentum and the nuclear spin moment. The details of the calculations of the hyperfine coupling tensor can be found in the Methods section.

**Figure 4:**
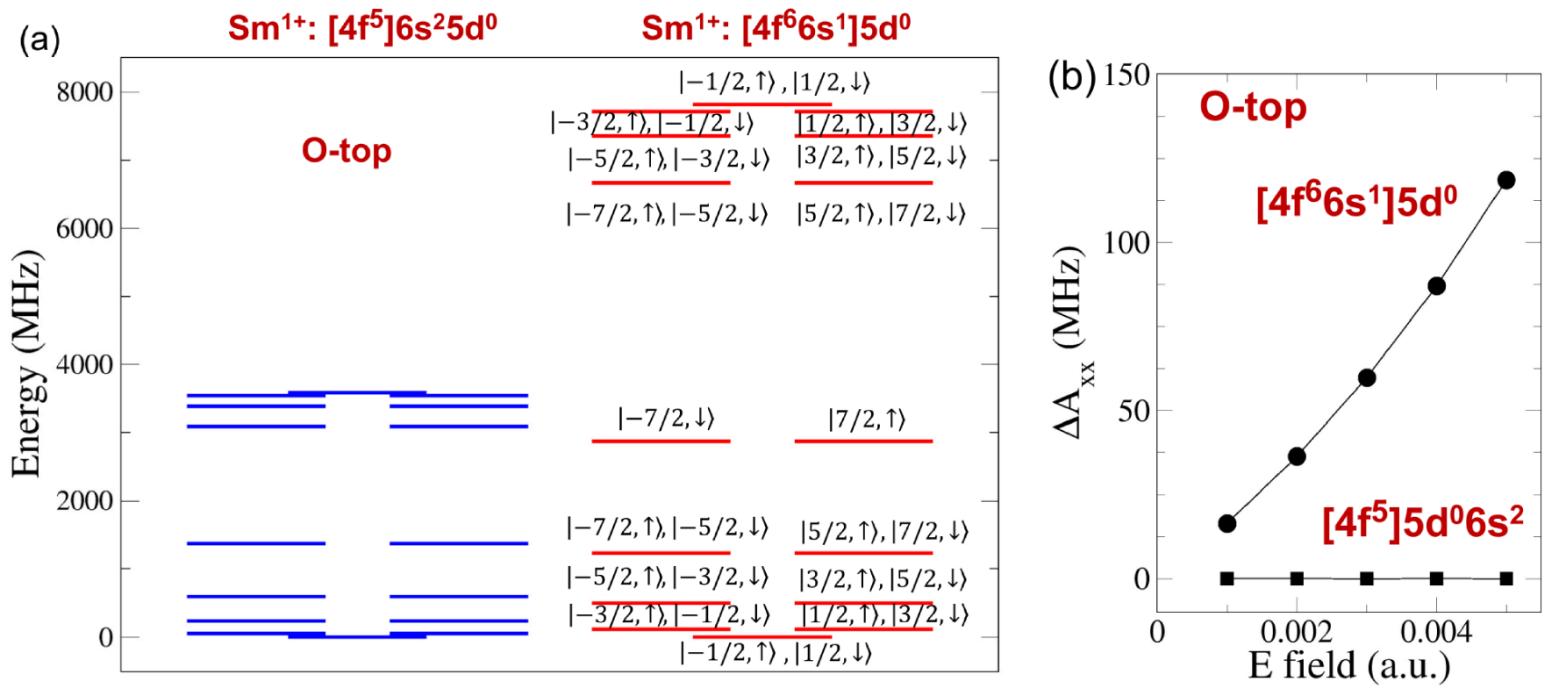
(a) Calculated low-energy electronic-nuclear spectra of the electronic configurations 4f^5^5d^0^6s^2^ and 4f^6^6s^1^5d^0^ for the O-top site. For each energy level, two dominant states |*M*_I_, *M*_S_⟩ are shown. The dominant states for each energy level for the configuration 4f^5^5d^0^6s^2^ are the same as those for the configuration 4f^6^6s^1^5d^0^. (b) A change of *A*_*xx*_ relative to the value at zero electric field as a function of a static electric field along the *z* axis for the configurations 4f^5^5d^0^6s^2^ (filled squares) and 4f^6^6s^1^5d^0^ (filled circles) for the O-top site.

Table 3 lists our calculated elements of the hyperfine coupling tensor for the ^147^Sm nucleus associated with the electronic ground Kramers doublet for the configuration 4f^5^5d^0^6s^2^ for the O-top and bridge sites. For this configuration, the hyperfine coupling tensor comes mostly from the paramagnetic spin–orbital term with a small contribution of the spin-dipole term. The Fermi contact contribution is negligible. For the O-top site, the *C*_4*v*_ symmetry enforces only nonzero diagonal elements with *A*_*xx*_ = *A*_*yy*_. Thus, only *A*_*zz*_ and *A*_0_ terms in eq 4 survive. The electronic in-plane magnetic anisotropy ensures |*A*_*xx*,*yy*_| ≫ |*A*_*zz*_| or |*A*_0_| ≫ |*A*_*zz*_|, which results in strong coupling between |*M*_I_, *M*_*S*_ = −1/2⟩ and |*M*_I_ −1, *M*_*S*_ = 1/2⟩ states. A small nuclear quadrupole interaction (see Table 4) does not affect this coupling much. The electronic-nuclear spectrum calculated including both hyperfine and nuclear quadrupole interactions is shown on the left-hand side of Figure 4a. The lowest and the highest levels are . The other electronic-nuclear levels are doubly degenerate. For example, the first-excited doublet consists of . Neighboring electronic-nuclear levels are separated by several tens to several thousands MHz. For the bridge site, the features of the electronic-nuclear level spectrum are similar to those for the O-top site.

**Table 3:** Calculated Elements of the Hyperfine Coupling Tensor in MHz for ^147^Sm at the O-Top and Bridge Sites for Three Active Spaces. *A*_1_ is Zero for All the Cases^a^

Site	Configuration	CASSCF	*A*_*xx*_	*A*_*xy*_	*A*_*xz*_	*A*_*yy*_	*A*_*yz*_	*A*_*zz*_
O-Top	[4f^5^]5d^0^6s^2^	CAS5-7	–897.3	0.0	0.0	–897.3	0.0	–224.6
O-Top	[4f^6^ 6s^1^]5d^0^	CAS7-8	–1954.4	0.0	0.0	–1954.4	0.0	–546.4
O-Top	[4f^5^5d^1^6s^1^]	CAS7-13	–111.4	0.0	0.0	–111.4	0.0	–3996.4
Bridge	[4f^5^]5d^0^6s^2^	CAS5-7	–834.4	586.0	0.0	–834.4	0.0	–140.1
Bridge	[4f^6^6s^1^]5d^0^	CAS7-8	–1546.1	1340.7	0.0	–1546.1	0.0	–201.3
Bridge	[4f^5^5d^1^6s^1^]	CAS7-13	–1767.4	–1464.5	0.0	–1767.4	0.0	–433.8

a Active orbitals are within brackets.

**Table 4:** Calculated Elements of the Nuclear Quadrupole Tensor in MHz for ^147^Sm^1+^ at the O-Top and Bridge Sites for Three Active Spaces

Site	Configuration	CASSCF	*P*_*xx*_	*P*_*xy*_	*P*_*xz*_	*P*_*yy*_	*P*_*yz*_	*P*_*zz*_
O-Top	[4f^5^]5d^0^6s^2^	CAS5-7	–1.73	0.00	0.00	–1.73	0.00	3.46
O-Top	[4f^6^6s^1^]5d^0^	CAS7-8	–1.67	0.00	0.00	–1.67	0.00	3.34
O-Top	[4f^5^5d^1^6s^1^]	CAS7-13	–4.69	0.00	0.00	–4.69	0.00	–9.38
Bridge	[4f^5^]5d^0^6s^2^	CAS5-7	–0.77	0.29	0.00	–0.77	0.00	1.54
Bridge	[4f^6^6s^1^]5d^0^	CAS7-8	–0.63	–0.39	0.00	–0.63	0.00	1.26
Bridge	[4f^5^5d^1^6s^1^]	CAS7-13	–0.54	3.99	0.00	–0.54	0.00	1.08

In the configuration 4f^5^5d^0^6s^2^, since the 6*s* orbital is doubly occupied (or the Fermi contact contribution is negligible), we expect that the effect of a static electric field on the hyperfine coupling tensor is negligible. Figure 4b shows a change of the element of the hyperfine coupling tensor, Δ*A*_*xx*_, as a function of an electric field along the *z* axis, for the O-top site. As expected, Δ*A*_*xx*_ is almost zero. For the O-top site, we examine Rabi oscillations induced by a time-dependent electric field applied along the *x* axis. In order for the frequency of the electric field to match the energy difference between the electronic-nuclear levels, a magnetic field is applied along the *z* axis. The electronic-nuclear levels are split by the nuclear Zeeman Hamiltonian  and electronic Zeeman Hamiltonian , where μ_B_ is Bohr magneton and *Ĥ*_*z*_ is the *z* component of the effective electronic spin operator. Here  is the *zz* component of the electronic *g* tensor, *g*_e_, for the electronic ground Kramers doublet which is calculated to be 0.9504. In the presence of a magnetic field of 2.0 T, the levels are split into individual |*M*_I_, *M*_S_⟩ levels. In this case, the electric field along the *x* axis can modulate the hyperfine interaction and the nuclear quadrupole interaction such that *A*_*xz*_ ≠ 0, *A*_*xx*_ ≠ *A*_*yy*_, *P*_*xz*_ ≠ 0, and *P*_*xx*_ ≠ *P*_*yy*_. This leads to Rabi oscillations between two |*M*_I_, *M*_S_⟩ levels whose quantum numbers differ by Δ*M*_I_ = ± 1 or Δ*M*_I_ = ± 2 while maintaining Δ*M*_S_ = 0, or Δ*M*_S_ = ± 1 with Δ*M*_I_ = 0. The latter can be observed by ESR-STM experiments. (Although Rabi oscillations are allowed between the levels whose quantum numbers differ by Δ*M*_I_ = ± 1 and Δ*M*_*S*_ = ± 1, they do not satisfy selection rules of spectroscopy techniques. Therefore, we do not consider them.) Calculated Rabi frequencies are of an order of 0.1 MHz (see Table S7), which is the same order of magnitude as those for the configuration 4f^6^5d^0^6s^2^. For the bridge site, calculated Rabi frequencies are enhanced compared to the O-top site and they are of up to an order of 10 MHz, when a time-independent electric field is applied along the *z* axis (see Tables S8 and S9).

Regarding the electronic configuration 4f^6^6s^1^5d^0^ (or equivalently 4f^6^(6s5d)^1^), according to Hund’s rule, the total angular momentum from the 4f^6^ shell is zero and the spin angular momentum from the 6s shell is 1/2. Thus, the total angular momentum from the configuration is speculated to be *J* = 1/2 in the electronic ground state. However, assignment of *J* to electronic excited multiplets is not valid due to mixtures of different atomic *J* multiplets in the low-lying electronic states, which is caused by the fact that the spin–orbit coupling is comparable to the crystal field (see Figure S4 and Table S10). For the O-top (bridge) site, the electronic ground Kramers doublet is well separated from the electronic first-excited Kramers doublet by 205 cm^–1^ (349 cm^–1^) which is several orders of magnitude larger than the hyperfine interaction and nuclear quadrupole interaction for the ^147^Sm nucleus.

Tables 3 and 4 list the elements of the hyperfine coupling tensor and nuclear quadrupole tensor for the ^147^Sm nucleus interacting with the electronic ground Kramers doublet in the configuration 4f^6^6s^1^5d^0^. The diagonal elements of the hyperfine coupling tensor are approximately twice as large as those in the configuration 4f^5^5d^0^6s^2^. This large increase in the hyperfine interaction arises from a large increase in the Fermi contact interaction between the electron spin density at the ^147^Sm nuclear position and the nuclear spin moment, which is consistent with the presence of an unpaired 6s orbital. The spin-dipole and paramagnetic spin–orbital terms are much less than the Fermi contact term. The characteristics of the electronic-nuclear levels are similar to those for the configuration 4f^5^5d^0^6s^2^. Neighboring electronic-nuclear levels are much farther separated than those for the configuration 4f^5^5d^0^6s^2^ (right-hand side of Figure 4a).

We discuss the effect of a static electric field on the hyperfine coupling tensor for the configuration 4f^6^6s^1^5d^0^. Figure 4b shows a change of the element of the hyperfine coupling tensor, Δ*A*_*xx*_, as a function of an electric field along the *z* axis, for the O-top site. We find that Δ*A*_*xx*_/*A*_*xx*_ is a few percents. The hyperfine Stark effect is large due to the large Fermi contact contribution caused by the unpaired 6*s* orbital.

**Figure 5:**
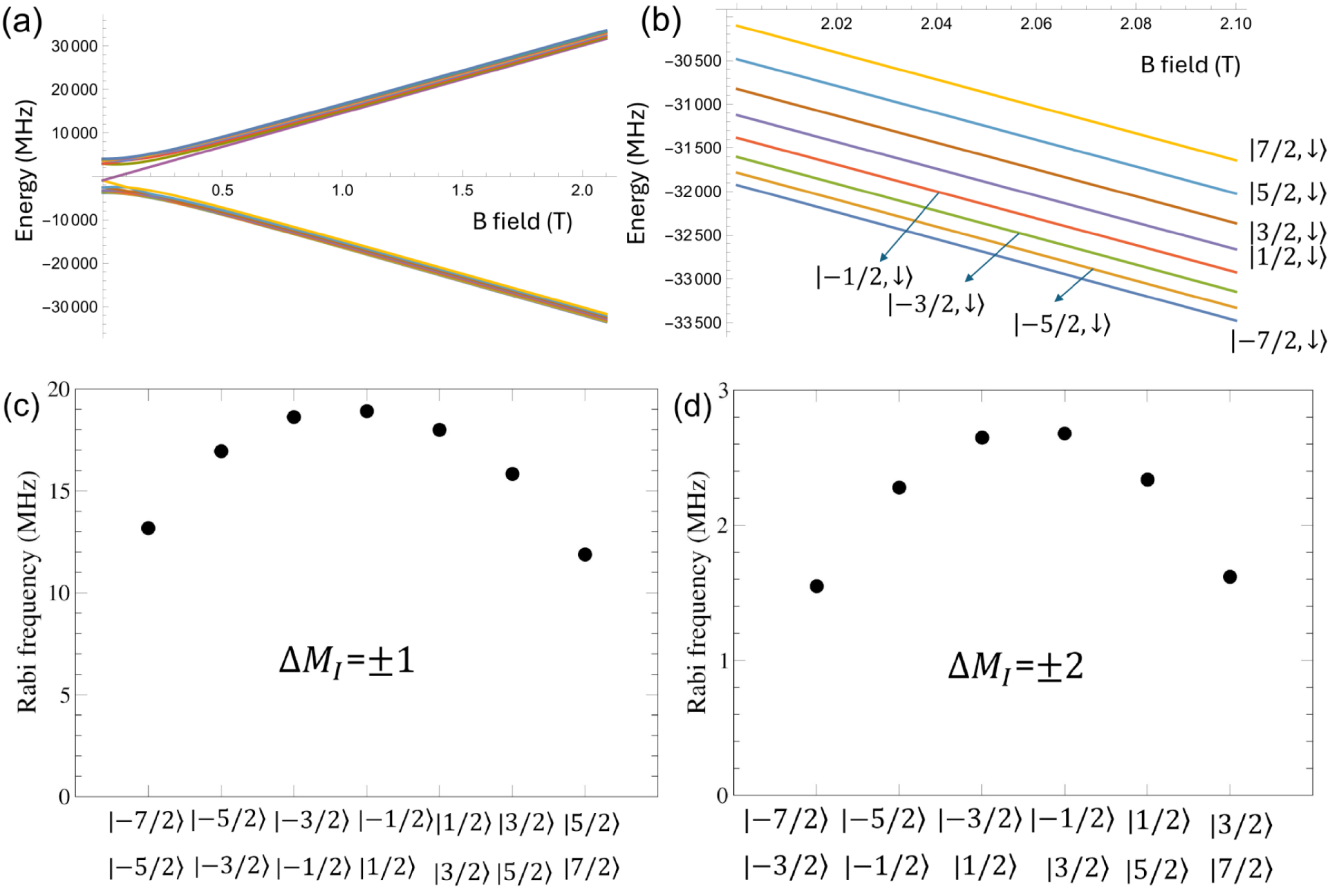
(a) Calculated Zeeman energy spectra of the electronic configuration 4f^6^6s^1^5d^0^ for the O-top site when a magnetic field is applied along the *z* axis. (b) Zoom-in of (a) near 2.0 T with labeling of eight low-lying electronic-nuclear states. Here down arrows in the states represent *M*_S_ = −1/2. (c),(d) Rabi frequencies associated with transitions between |*M*_I_, *M*_S_⟩ levels (among the eight levels) whose quantum numbers differ by Δ*M*_I_ = ±1 or Δ*M*_I_ = ±2 (and Δ*M*_S_ = 0) with *B*_*z*_ = 2.0 T and *E*_*x*_ = 0.002 au (≈1 GV/m) for the configuration 4f^6^6s^1^5d^0^ at the O-top site. Here only *M*_I_ values of two electronic-nuclear levels involved with the transitions are listed.

For the O-top site, we investigate Rabi oscillations induced by a time-dependent electric field applied along the *x* axis. Figure 5a,b shows the electronic-nuclear levels split by the nuclear Zeeman Hamiltonian and electronic Zeeman Hamiltonian, where the *zz* component of the electronic *g* tensor for the electronic ground Kramers doublet is calculated to be 2.2144. Figure 5c,d shows calculated Rabi frequencies for the eight low-lying levels for the O-top site in the presence of a magnetic field of 2.0 T. (Additional Rabi frequencies can be found in Table S11). The Rabi frequencies are several to several tens MHz which are at least 1 order of magnitude larger than those for the configuration 4f^5^5d^0^6s^2^ for the O-top site. For the bridge site, Rabi frequencies induced by a time-dependent electric along the *z* axis can be found in Tables S12 and S13.

**Figure 6:**
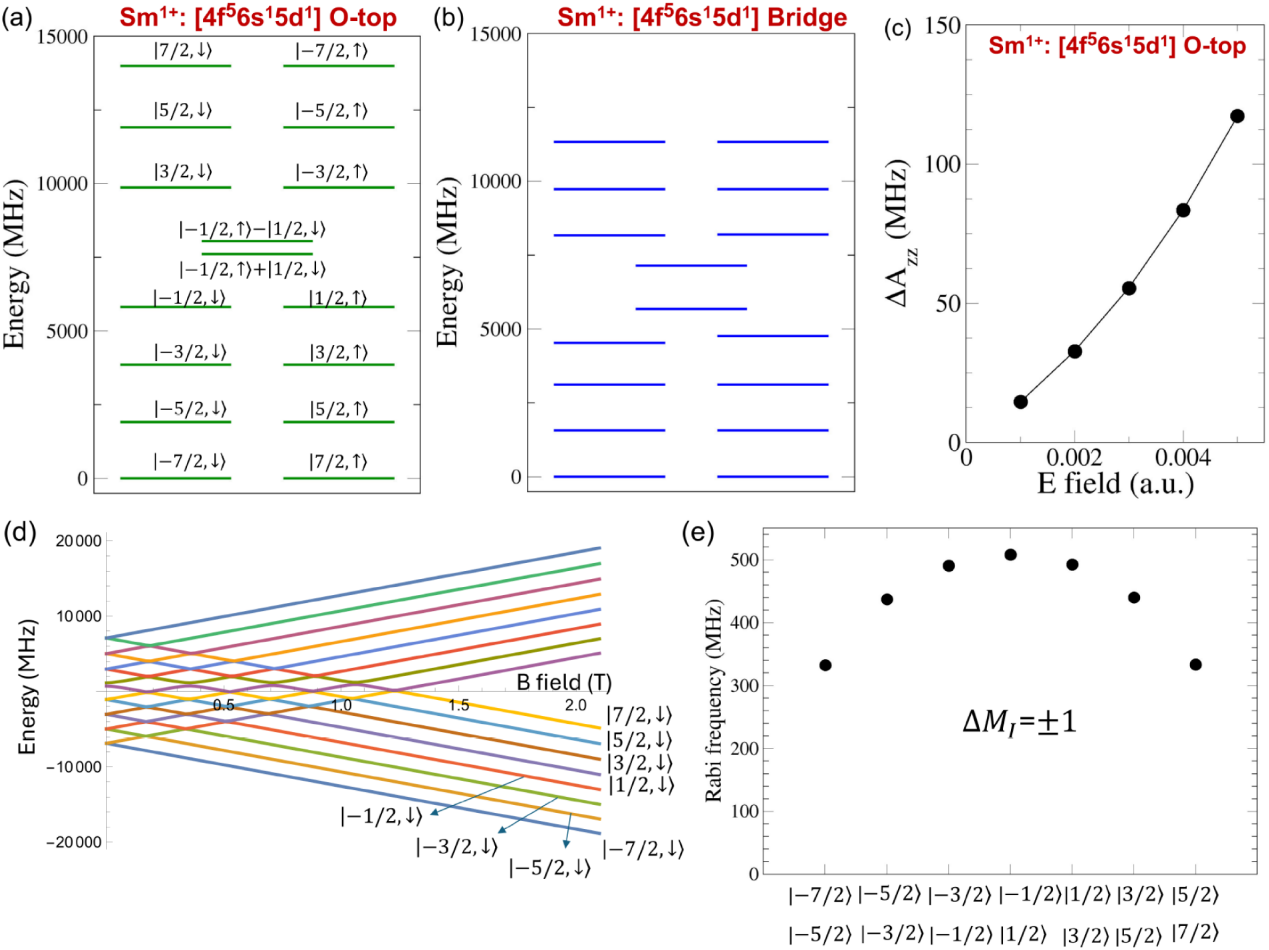
(a)-(b) Calculated low-energy electronic-nuclear spectra of the electronic configuration 4f^5^5d^1^6s^1^ for the O-top and bridge sites. For the O-top site, the electronic-nuclear levels |*M*_I_, *M*_S_⟩ are specified, where the arrows up and down indicate *M*_S_ = ±1/2. For the bridge site, the electronic-nuclear levels consist of highly mixed |*M*_I_, *M*_S_⟩ levels. (c) A change of *A*_*xx*_ relative to the value at zero electric field as a function of an electric field along the *z* axis for the configuration 4f^5^5d^1^6s^1^. (d) Zeeman diagram of the electronic-nuclear levels and (e) Rabi frequencies associated with transitions between |*M*_I_, *M*_S_⟩ levels (among the eight low-lying levels) whose quantum numbers differ by Δ*M*_I_ = ±1 and Δ*M*_S_ = 0 with *B*_*z*_ = 2.0 T and *E*_*x*_ = 0.002 au for the configuration 4f^1^5d^1^6s^1^ at the O-top site. Only *M*_I_ levels are indicated.

For the electronic configuration 4f^5^5d^1^6s^1^, several low-lying electronic energies for the O-top and bridge sites are shown in Table S14. Similarly to the other configurations, effective *J* values cannot be assigned to the electronic states since the crystal field is comparable to the spin–orbit coupling. For the O-top (bridge) site, the electronic ground Kramers doublet is well separated from the electronic first-excited doublet by 146 cm^–1^ (191 cm^–1^). Therefore, we can assign an effective electron spin *S*_eff_ = 1/2 for the electronic ground Kramers doublet. As discussed earlier for the other electronic configurations, this separation of the electronic energies is much larger than the hyperfine interaction and nuclear quadrupole interaction energies (see Tables 3 and 4). For this configuration, the Fermi contact contribution to the hyperfine interaction is much larger than the spin-dipole and paramagnetic spin–orbital terms. Interestingly, the features of the hyperfine interaction for the ^147^Sm nucleus for the O-top site are completely different from those for the bridge site (see Table 3). In the former, |*A*_*xx*,*yy*_| ≪ |*A*_*zz*_|, while in the latter, |*A*_*xx*,*yy*_| ≫ |*A*_*zz*_|. As shown in Figure 6a, for the O-top site, all the electronic-nuclear levels are doubly degenerate except for the middle two levels such as . Since *A*_*zz*_ is negative and dominant, the lowest ground doublet consists of , while the highest doublet arises mainly from  with a tiny contribution from . For the bridge site, the spectrum of the electronic-nuclear levels consists of several low-energy and high-energy quasi-doublets and two separate levels in the middle (Figure 6b). The electronic-nuclear levels comprise strongly mixed |*M*_I_, *M*_S_⟩ levels.

Since the Fermi contact contribution is large, we also expect a large hyperfine Stark effect in the configuration 4f^5^5d^1^6s^1^, similarly to the configuration 4f^6^6s^1^5d^0^. Figure 6c shows a change of *A*_*zz*_ as a function of a static electric field applied along the *z* axis for the O-top site. We find that Δ*A*_*zz*_/*A*_*zz*_ is a few percents in the range of the considered electric field.

Figure 6d shows a Zeeman diagram of the electronic-nuclear levels for the O-top site in the configuration 4f^5^5d^1^6s^1^, where the *zz* component of the electronic *g* tensor for the electronic ground Kramers doublet is calculated to be 0.8523. We examine Rabi oscillations between eight lowest |*M*_I_, *M*_S_⟩ levels whose quantum numbers differ by Δ*M*_I_ = ± 1 and Δ*M*_S_ = 0 for the O-top site with *B*_*z*_ = 2.0 T and *E*_*x*_ = 0.002 au, finding that Rabi frequencies are on the order of 100 MHz (see Figure 6e). This is 1 order of magnitude larger than that for the configuration 4f^6^6s^1^5d^0^. Additional Rabi frequencies for the O-top site and the bridge site can be found in Tables S15–S17.

We now compare our calculated result for the Sm^1+^ configuration 4f^6^6s^1^5d^0^ with the experimental data.^20^ In this experiment, the first-excited electronic state appeared at 38 meV (=306.5 cm^–1^) for the bridge site, whereas the first-excited and second-excited electronic states appeared at 26.2 meV (=209.7 cm^–1^) and 89 meV (=717.8 cm^–1^) for the O-top site, respectively. These experimental excitation energies are in good agreement with our calculated energies which are 348.7 cm^–1^, 204.7 cm^–1^, and 655.6 cm^–1^ for the bridge site and O-top site, respectively. In addition, the experimental electron spin resonance transitions from the hyperfine coupling appear around 30 GHz at the in-plane magnetic field of 0.455 T. Using our calculated *g*-tensor and hyperfine coupling tensor, we find that the spectrum occurs around 40 GHz, which is in reasonable agreement with the experimental data.

## Conclusions

We have investigated the electronic structure and electronic-nuclear and nuclear level spectra for a ^147^Sm nucleus for a Sm adatom on a MgO substrate by using the multiconfigurational *ab initio* method, considering two charge states and several electronic configurations including 5*d* and 6*s* valence shells for two adsorption sites. For a Sm adatom on MgO, the crystal field is comparable to the spin–orbit coupling. In this case, effective *J* values cannot be assigned to the electronic multiplets. Therefore, caution needs to be exercised for fitting experimental data to effective models. Regarding a neutral charge state, we examined the 4f^6^5d^0^6s^2^ and 4f^5^5d^1^6s^2^ configurations. For both configurations, the hyperfine interaction for the ^147^Sm nucleus is absent in the electronic ground state, and the nuclear levels for the ^147^Sm nucleus are split by the nuclear quadrupole interaction. We found that a static electric field can induce a significant quadrupole Stark effect especially for the bridge site, and that a time-dependent electric field applied along the *z* axis (*x* axis) can induce Rabi oscillations with frequencies of an order of 0.1 MHz (1 MHz) for the bridge (O-top) site. Regarding a singly charged state, we studied the 4f^5^5d^0^6s^2^, 4f^6^6s^1^5d^0^, and 4f^5^5d^1^6s^1^ configurations. For the 4f^6^6s^1^5d^0^ and 4f^5^5d^1^6s^1^ configurations, we showed a strong hyperfine Stark effect due to the large Fermi contact contribution induced by an unpaired 6*s* orbital, which greatly facilitates utilization and manipulation of the atomic-size qubits. This result can be generalized to other rare-earth adatoms on MgO or other substrates: a strong hyperfine Stark effect is expected for rare-earth adatoms with the electronic configurations 4f^*n*^6s^1^5d^0^ and 4*f*^*n*–1^5d^1^6s^1^. We also computed Rabi oscillations with frequencies of an order of 1–100 MHz induced by a time-dependent electric field applied along the *x* axis for the O-top site. Our findings can be observed by ESR-STM experiments^13,14^ or NMR-STM experiments.^3^

## Computational Methods

The structures of the three adsorption sites (O-top, bridge, Mg-top) for a neutral Sm adatom on a MgO(001) surface are obtained within density-functional theory (DFT) by using the VASP code.^27,28^ We use the Perdew–Burke–Ernzerhof (PBE) generalized gradient approximation^29^ for the exchange-correlation energy functional with projector-augmented-wave (PAW) pseudopotentials.^30,31^ Spin–orbit coupling is included self-consistently, and van der Waals interaction is included using the DFT-D3 correction method by Grimme et al.^32^ The kinetic energy cutoff is 400 eV. For the O-top and Mg-top sites, we consider a supercell consisting of a Sm adatom on an 8 × 8 × 4 MgO substrate with a vacuum layer of over 20 Å, and relax the atomic coordinates of the supercell (with the bottom two MgO layers fixed to the experimental lattice constant) until the residual force is less than 0.01 eV/Å. Here 4 × 4 × 1 *k*-points are sampled. The distance between the Sm adatom and the O atom (Mg atom) right below the Sm adatom is 2.138 Å (3.09 Å). For the quantum chemistry calculations, we carve out a cluster of 25 Mg and 25 O atoms near the Sm adatom from the DFT-relaxed structure. For the bridge site, we consider a supercell consisting of a Sm atom on a 6 × 6 × 4 MgO substrate with 5 × 5 × 1 *k*-points and a vacuum layer of over 20 Å, and relax the atomic coordinates with the bottom two MgO layers fixed, similarly to the O-top case. The closest distance between the Sm adatom and the O atoms is 2.032 Å. For the quantum chemistry calculations, we use a cluster of 16 Mg and 16 O atoms near the Sm adatom from the DFT-relaxed structure.

Multiconfigurational *ab initio* calculations are carried out for the aforementioned clusters using the Molcas/OpenMolcas quantum chemistry code.^33,34^ Scalar relativistic effects are included on the basis of the Douglas-Kroll-Hess (DKH) Hamiltonian^35,36^ using relativistically contracted atomic natural orbital (ANO-RCC) basis sets.^37,38^ Polarized valence triple-ζ quality (ANO-RCC-VTZP) is used for the Sm atom and polarized valence double-ζ quality (ANO-RCC-VDZP) is used for the Mg and O atoms.

The electronic structure is calculated in two steps. First, for each electronic configuration and each adsorption site, in the absence of spin–orbit interaction, one or two spin multiplicities are considered and spin-free eigenstates are computed using state-averaged CASSCF method. For the configurations [4f^6^]5d^0^6s^2^ and [4f^5^5d^1^]6s^2^, both *S* = 3 and *S* = 2 are included in the calculations. For the configuration [4f^6^6s^1^]5d^0^, both *S* = 7/2 and *S* = 5/2 are considered. For the configurations [4f^5^5d^1^6s^1^] and [4f^5^]5d^0^6s^2^, *S* = 7/2 and *S* = 5/2 are included, respectively. Considering more than one spin multiplicities does not change the results much. Second, spin–orbit interaction is included for the spin-free eigenstates within the atomic mean-field approximation^39^ using the RASSI method.^21^

The nuclear quadrupole interaction for the ^147^Sm nucleus is computed for the electronic ground singlet or doublet using the method discussed in ref (23). The hyperfine coupling tensor for the ^147^Sm nucleus associated with the electronic ground Kramers doublet is computed in the crystal coordinates by treating the hyperfine interaction nonrelativistically^23,26^ for the [4f^5^]5d^0^6s^2^ configuration and by treating the hyperfine interaction relativistically^40^ for the [4f^6^6s^1^]5d^0^ and [4f^5^5d^1^6s^1^] configurations. The relativistic treatment of the hyperfine interaction is needed due to a large Fermi contact contribution induced by the unpaired 6s orbital. For the calculation of the relativistic hyperfine interaction, we use the implementation discussed in ref (40), which is based on the DKH theory.^35,36,41,42^ Although the signs of the elements of the hyperfine coupling tensor cannot be determined from the implementations in refs (23, 26, and 40), they can be identified as follows. For the Sm nucleus, the Fermi contact term, spin-dipole term, and paramagnetic spin–orbital term are all added up to form the hyperfine coupling tensor. Therefore, we focus on the signs of the tensor elements from the Fermi contact term. Since the Fermi contact contribution is isotropic, the signs of all elements must be the same. The Fermi contact contribution to the nonrelativistic hyperfine coupling constant^43^ can be written as
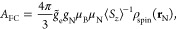
5where *g*_e_ is a free electron *g* factor. As shown in eq 5, the sign of the Fermi contact contribution is determined by the signs of *g*_N_ and the electron spin density ρ_spin_ at the nuclear position **r**_N_. From our calculations, we find that the Mulliken spin population of the 6*s* orbital at the Sm site is positive and the sign of *g*_N_ for the Sm nucleus is negative. Therefore, the overall sign of the Fermi contact term is negative and the overall sign of the hyperfine coupling tensor for the Sm nucleus is negative. Note that our calculations of the hyperfine coupling tensor in either nonrelativistic or relativistic case are performed using the multiconfigurational *ab initio* method.

## Supporting Information Available

Low-lying electronic energy levels for all the configurations; crystal-field parameters for the lowest electronic multiplet for the configuration [4f^5^]5d^0^6s^2^ (CAS5-7) at the bridge site; Rabi frequencies associated with transitions between nuclear levels or electronic-nuclear levels for the electronic configuration [4f^5^5d^1^]6s^2^ (CAS6-12), [4f^5^]5d^0^s^2^ (CAS5-7), [4f^6^6s^1^]5d^0^ (CAS7-8), and [4f^5^5d^1^6s^1^] (CAS7-13) at the O-top and bridge sites (PDF)

## References

[ref1] Gambardella P.; Rusponi S.; Veronese M.; Dhesi S. S.; Grazioli C.; Dallmeyer A.; Cabria I.; Zeller R.; Dederichs P. H.; Kern K.; Carbone C.; Brune H. Giant Magnetic Anisotropy of Single Cobalt Atoms and Nanoparticles. Science 2003, 300, 1130–1133. 10.1126/science.1082857. 12750516

[ref2] Baltic R.; Donati F.; Singha A.; Wäckerlin C.; Dreiser J.; Delley B.; Pivetta M.; Rusponi S.; Brune H. Magnetic properties of single rare-earth atoms on graphene/Ir(111). Phys. Rev. B 2018, 98, 024412 10.1103/PhysRevB.98.024412.

[ref3] Yang K.; Willke P.; Bae Y.; Ferrón A.; Lado J. L.; Ardavan A.; Fernández-Rossier J.; Heinrich A. J.; Lutz C. P. Electrically controlled nuclear polarization of individual atoms. Nat. Nanotechnol. 2018, 13, 1120–1125. 10.1038/s41565-018-0296-7. 30397285

[ref4] Willke P.; Bae Y.; Yang K.; Lado J. L.; Ferrón A.; Choi T.; Ardavan A.; Fernández-Rossier J.; Heinrich A. J.; Lutz C. P. Hyperfine interaction of individual atoms on a surface. Science 2018, 362, 336–339. 10.1126/science.aat7047. 30337408

[ref5] Pivetta M.; Patthey F.; Di Marco I.; Subramonian A.; Eriksson O.; Rusponi S.; Brune H. Measuring the Intra-Atomic Exchange Energy in Rare-Earth Adatoms. Phys. Rev. X 2020, 10, 031054 10.1103/PhysRevX.10.031054.

[ref6] Donati F. Correlation between Electronic Configuration and Magnetic Stability in Dysprosium Single Atom Magnets. Nano Lett. 2021, 21, 8266–8273. 10.1021/acs.nanolett.1c02744. 34569802

[ref7] Ternes M.; Lutz C. P.; Heinrich A. J.; Schneider W.-D. Sensing the Spin of an Individual Ce Adatom. Phys. Rev. Lett. 2020, 124, 167202 10.1103/PhysRevLett.124.167202. 32383899

[ref8] Singha A. Mapping Orbital-Resolved Magnetism in Single Lanthanide Atoms. ACS Nano 2021, 15, 16162–16171. 10.1021/acsnano.1c05026. 34546038

[ref9] Chen Y.; Bae Y.; Heinrich A. J. Harnessing the Quantum Behavior of Spins on Surfaces. Adv. Mater. 2023, 35, 2107534 10.1002/adma.202107534. 34994026

[ref10] Reale S.; Singha A.; Ahmed S. L.; Krylov D.; Colazzo L.; Wolf C.; Casari C. S.; Barla A.; Fernandes E.; Patthey F.; Pivetta M.; Rusponi S.; Brune H.; Donati F. Erbium and thulium on MgO(100)/Ag(100) as candidates for single atom qubits. Phys. Rev. B 2023, 107, 045427 10.1103/PhysRevB.107.045427.

[ref11] Reale S.; Hwang J.; Oh J.; Brune H.; Heinrich A. J.; Donati F.; Bae Y. Electrically driven spin resonance of 4f electrons in a single atom on a surface. Nat. Commun. 2024, 15, 5289 10.1038/s41467-024-49447-y. 38902242 PMC11190280

[ref12] Wang Y.; Chen Y.; Bui H. T.; Wolf C.; Haze M.; Mier C.; Kim J.; Choi D.-J.; Lutz C. P.; Bae Y.; et al. An atomic-scale multi-qubit platform. Science 2023, 382, 87–92. 10.1126/science.ade5050. 37797000

[ref13] Baumann S.; Paul W.; Choi T.; Lutz C. P.; Ardavan A.; Heinrich A. J. Electron paramagnetic resonance of individual atoms on a surface. Science 2015, 350, 417–420. 10.1126/science.aac8703. 26494753

[ref14] Paul W.; Baumann S.; Lutz C. P.; Heinrich A. J. Generation of constant-amplitude radio-frequency sweeps at a tunnel junction for spin resonance STM. Rev. Sci. Instrum. 2016, 87, 074703 10.1063/1.4955446. 27475577

[ref15] Serrano D.; Kuppusamy S. K.; Heinrich B.; Fuhr O.; Hunger D.; Ruben M.; Goldner P. Ultra-narrow optical linewidths in rare-earth molecular crystals. Nature 2022, 603, 241–246. 10.1038/s41586-021-04316-2. 35264757

[ref16] Asaad S.; Mourik V.; Joecker B.; Johnson M. A. I.; Baczewski A. D.; Firgau H. R.; Mądzik M. T.; Schmitt V.; Pla J. J.; Hudson F. E.; et al. Coherent electrical control of a single high-spin nucleus in silicon. Nature 2020, 579, 205–209. 10.1038/s41586-020-2057-7. 32161384

[ref17] Kane B. E. A silicon-based nuclear spin quantum computer. Nature 1998, 393, 133–137. 10.1038/30156.

[ref18] Thiele S.; Balestro F.; Ballou R.; Klyatskaya S.; Ruben M.; Wernsdorfer W. Electrically driven nuclear spin resonance in single-molecule magnets. Science 2014, 344, 1135–1138. 10.1126/science.1249802. 24904159

[ref19] Singha A.; Baltic R.; Donati F.; Wäckerlin C.; Dreiser J.; Persichetti L.; Stepanow S.; Gambardella P.; Rusponi S.; Brune H. 4*f* occupancy and magnetism of rare-earth atoms adsorbed on metal substrates. Phys. Rev. B 2017, 96, 224418 10.1103/PhysRevB.96.224418.

[ref20] Czap G.; Noh K.; Velasco J. Jr.; Macfarlane R. M.; Brune H.; Lutz C. P. Direct electrical access to the spin manifolds of individual monovalent lanthanide atoms. arXiv 2024. 10.1021/acsnano.4c14327 PMC11781023 39808209

[ref21] Malmqvist P.-Å.; Roos B. O.; Schimmelpfennig B. The Restricted Active Space (RAS) State Interaction Approach with Spin-Orbit Coupling. Chem. Phys. Lett. 2002, 357, 230–240. 10.1016/S0009-2614(02)00498-0.

[ref22] Donati F.; Singha A.; Stepanow S.; Wäckerlin C.; Dreiser J.; Gambardella P.; Rusponi S.; Brune H. Magnetism of Ho and Er Atoms on Close-Packed Metal Surfaces. Phys. Rev. Lett. 2014, 113, 237201 10.1103/PhysRevLett.113.237201. 25526151

[ref23] Wysocki A. L.; Park K. Nature of Hyperfine Interactions in TbPc2 Single-Molecule Magnets: Multiconfigurational Ab Initio Study. Inorg. Chem. 2020, 59, 2771–2780. 10.1021/acs.inorgchem.9b03136. 32072814

[ref24] Ono M.; Ishihara J.; Sato G.; Ohno Y.; Ohno H. Coherent Manipulation of Nuclear Spins in Semiconductors with an Electric Field. Appl. Phys. Express 2013, 6, 033002 10.7567/APEX.6.033002.

[ref25] Rinehart J. D.; Long J. R. Exploiting single-ion anisotropy in the design of f-element single-molecule magnets. Chem. Sci. 2011, 2, 2078–2085. 10.1039/c1sc00513h.

[ref26] Sharkas K.; Pritchard B.; Autschbach J. Effects from Spin-Orbit Coupling on Electron-Nucleus Hyperfine Coupling Calculated at the Restricted Active Space Level for Kramers Doublets. J. Chem. Theory Comput. 2015, 11, 538–549. 10.1021/ct500988h. 26580911

[ref27] Kresse G.; Furthmüller J. Efficient iterative schemes for ab initio total-energy calculations using a plane-wave basis set. Phys. Rev. B 1996, 54, 11169–11186. 10.1103/PhysRevB.54.11169. 9984901

[ref28] Kresse G.; Furthmüller J. Efficiency of ab-initio total energy calculations for metals and semiconductors using a plane-wave basis set. Comput. Mater. Sci. 1996, 6, 15–50. 10.1016/0927-0256(96)00008-0. 9984901

[ref29] Perdew J. P.; Burke K.; Ernzerhof M. Generalized Gradient Approximation Made Simple. Phys. Rev. Lett. 1996, 77, 3865–3868. 10.1103/PhysRevLett.77.3865. 10062328

[ref30] Blöchl P. E. Projector augmented-wave method. Phys. Rev. B 1994, 50, 17953–17979. 10.1103/PhysRevB.50.17953. 9976227

[ref31] Kresse G.; Joubert D. From ultrasoft pseudopotentials to the projector augmented-wave method. Phys. Rev. B 1999, 59, 1758–1775. 10.1103/PhysRevB.59.1758.

[ref32] Grimme S.; Antony J.; Ehrlich S.; Krieg H. A consistent and accurate ab initio parametrization of density functional dispersion correction (DFT-D) for the 94 elements H-Pu. J. Chem. Phys. 2010, 132, 154104 10.1063/1.3382344. 20423165

[ref33] Aquilante F.; Autschbach J.; Carlson R. K.; Chibotaru L. F.; Delcey M. G.; De Vico L.; Galván I. F.; Ferré N.; Frutos L. M.; Gagliardi L.; et al. Molcas 8: New Capabilities for Multiconfigurational Quantum Chemical Calculations across the Periodic Table. J. Comput. Chem. 2016, 37, 506–541. 10.1002/jcc.24221. 26561362

[ref34] Galván I. F.; Vacher M.; Alavi A.; Angeli C.; Aquilante F.; Autschbach J.; Bao J. J.; Bokarev S.I.; Bogdanov N. A.; Carlson R. K. OpenMolcas: From Source Code to Insight. J. Chem. Theory Comput. 2019, 15, 5925–5964. 10.1021/acs.jctc.9b00532. 31509407

[ref35] Douglas M.; Kroll N. M. Quantum Electrodynamical Corrections to the Fine Structure of Helium. Ann. Phys. 1974, 82, 89–155. 10.1016/0003-4916(74)90333-9.

[ref36] Hess B. A. Relativistic Electronic-Structure Calculations Employing a Two-Component No-Pair Formalism with External-Field Projection Operators. Phys. Rev. A 1986, 33, 3742–3748. 10.1103/PhysRevA.33.3742. 9897114

[ref37] Roos B. O.; Lindh R.; Malmqvist P.-Ã.; Veryazov V.; Widmark P.-O. Main Group Atoms and Dimers Studied with a New Relativistic ANO Basis Set. J. Phys. Chem. A 2004, 108, 2851–2858. 10.1021/jp031064+.

[ref38] Roos B. O.; Lindh R.; Malmqvist P.-Ã.; Veryazov V.; Widmark P.-O.; Borin A. C. New Relativistic Atomic Natural Orbital Basis Sets for Lanthanide Atoms with Applications to the Ce Diatom and LuF3. J. Phys. Chem. A 2008, 112, 11431–11435. 10.1021/jp803213j. 18928264

[ref39] Heß B. A.; Marian C. M.; Wahlgren U.; Gropen O. A Mean-Field Spin-Orbit Method Applicable to Correlated Wavefunctions. Chem. Phys. Lett. 1996, 251, 365–371. 10.1016/0009-2614(96)00119-4.

[ref40] Wysocki A. L.; Park K. Relativistic Douglas–Kroll–Hess calculations of hyperfine interactions within first-principles multireference methods. J. Chem. Phys. 2024, 160, 224102 10.1063/5.0208851. 38856053

[ref41] Wolf A.; Reiher M.; Hess B. A. The generalized Douglas-Kroll transformation. J. Chem. Phys. 2002, 117, 9215–9226. 10.1063/1.1515314. 15267790

[ref42] Reiher M.; Wolf A. Exact decoupling of the Dirac Hamiltonian. I. General theory. J. Chem. Phys. 2004, 121, 2037–2047. 10.1063/1.1768160. 15260757

[ref43] Munzarová M.; Kaupp M. A Critical Validation of Density Functional and Coupled-Cluster Approaches for the Calculation of EPR Hyperfine Coupling Constants in Transition Metal Complexes. J. Phys. Chem. A 1999, 103, 9966–9983. 10.1021/jp992303p.

[ref44] Boerner T. J.; Deems S.; Furlani T. R.; Knuth S. L.; Towns J. ACCESS: Advancing Innovation: NSF’s Advanced Cyberinfrastructure Coordination Ecosystem: Services & Support. In PEARC’23: Practice and Experience in Advanced Research Computing 2023: Computing for the Common Good. ACM, New York, NY, USA, 2023; 173–176.

