# Supplementary material for: Electrical Control of the Nuclear Spin States of Rare-Earth Adatoms
Source: ACS Nano. 2025 Apr 21;19(17):16372–82. doi: 10.1021/acsnano.4c16416 (PMC12060648; doi:10.1021/acsnano.4c16416)
Supplement: Supplementary file 1 — nn4c16416_si_001.pdf [file nn4c16416_si_001.pdf]

Supporting Information:

Electrical control of the nuclear spin states of rare-earth adatoms

Homa Karimi,[1] Aleksander L. Wysocki,[2] and Kyungwha Park[1,*]

[1] *Department of Physics, Virginia Tech, Blacksburg, Virginia, 24061 USA*

[2] *Department of Physics and Astronomy,*
University of Nebraska at Kearney, Kearney, Nebraska 68849 USA

(Dated: February 23, 2025)

* kyungwha@vt.edu

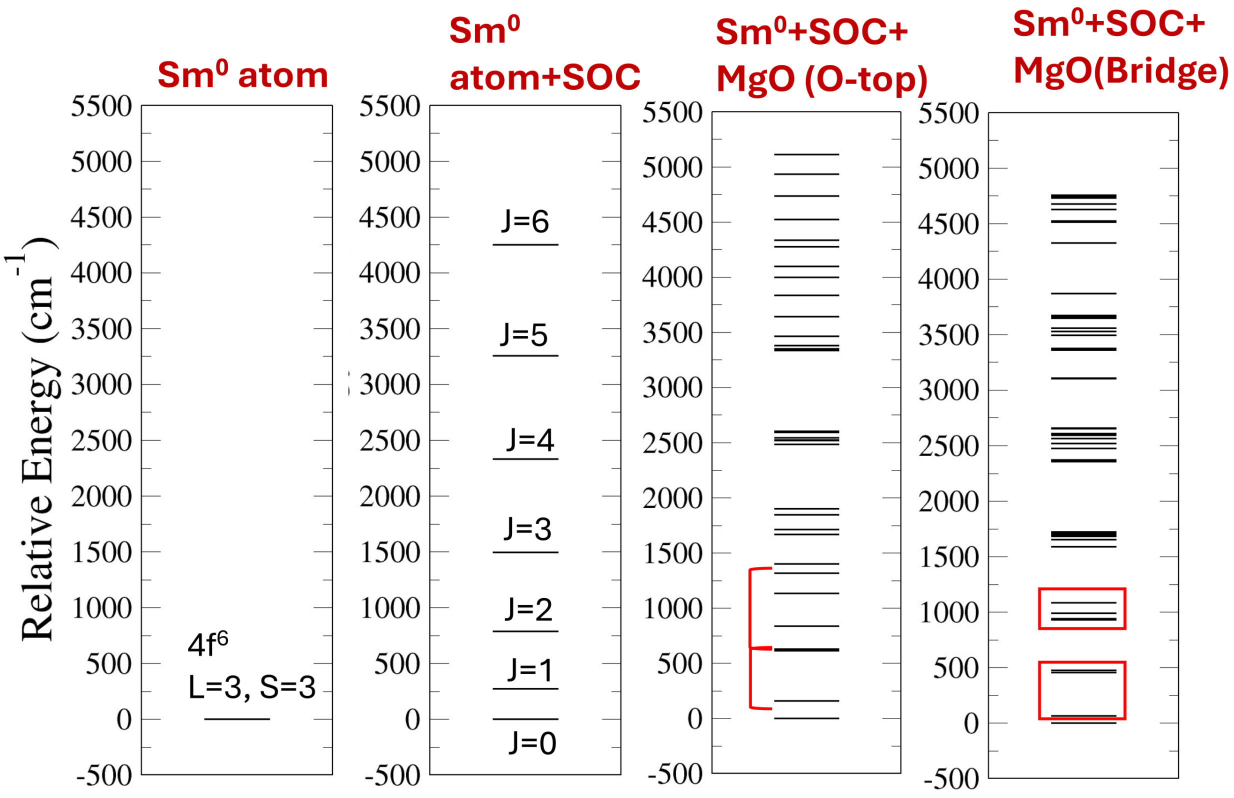

FIG. S1. Electronic structures of a neutral isolated Sm atom and a neutral Sm adatom on MgO for the electronic configuration $[4f^6]5d^06s^2$ (CAS6-7) at the O-top and bridge sites. Since spin-orbit coupling is comparable to the crystal field, effective J values cannot be assigned to excited electronic multiplets.

TABLE S1. Low-lying electronic energies of the neutral Sm case for the electronic configuration $[4f^6]5d^0 6s^2$ (CAS6-7) at the O-top and bridge sites as well as for an isolated Sm^0 atom

isolated atom		O-top	Bridge
Energy (cm^{-1})	J	Energy (cm^{-1})	Energy (cm^{-1})
0	0	0	0
274.0	1	159.1	64.9
274.0	1	159.1	457.5
274.0	1	614.6	474.9
790.0	2	628.4	929.5
790.0	2	837.4	930.2
790.0	2	1134.7	939.5
790.0	2	1134.7	992.6
790.0	2	1317.9	1082.6

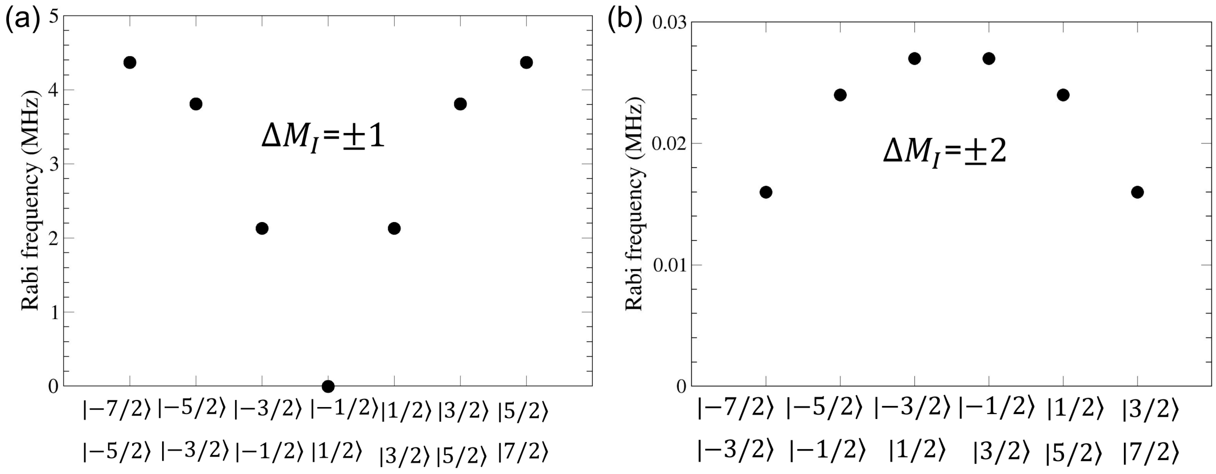

FIG. S2. (a),(b) Rabi frequencies associated with transitions between the nuclear levels whose quantum numbers differ by $\Delta M_I = \pm 1$ or ± 2 for the electronic configuration $[4f^5 5d^1]6s^2$ (CAS6-12) at the O-top site. A time-dependent electric field whose amplitude is 0.002 a.u. is applied along the x axis in the presence of a magnetic field of 0.5 T along the z axis.

TABLE S2. Low-lying electronic energies of the neutral Sm case for the electronic configuration $[4f^5 5d^1]6s^2$ (CAS6-12) at the O-top and bridge sites

O-top Energy (cm^{-1})	Bridge Energy (cm^{-1})
0	0
23.5	45.7
23.7	338.6
43.7	502.2
207.6	575.7
598.0	765.4
844.5	769.9
844.7	1058.6
900.5	1170.2
1019.5	1172.3
1019.5	1314.5

TABLE S3. Nuclear eigenstates for the electronic ground state of the electronic configuration $[4f^5 5d^1]6s^2$ (CAS6-12) at the bridge site when a magnetic field of 0.5 T is applied along the z axis. Only coefficients greater than 0.1 are shown.

State #	Eigenstate
1	$-0.16i|-\frac{7}{2}\rangle - 0.59|-\frac{3}{2}\rangle + 0.71i|\frac{1}{2}\rangle + 0.34|\frac{5}{2}\rangle$
2	$-0.37i|-\frac{5}{2}\rangle - 0.73|-\frac{1}{2}\rangle + 0.56i|\frac{3}{2}\rangle + 0.14|\frac{7}{2}\rangle$
3	$(-0.16+0.40i)|-\frac{7}{2}\rangle + (0.55+0.22i)|-\frac{3}{2}\rangle + (-0.11+0.27i)|\frac{1}{2}\rangle + (0.57+0.22i)|\frac{5}{2}\rangle$
4	$-0.66i|-\frac{5}{2}\rangle - 0.21|-\frac{1}{2}\rangle - 0.62i|\frac{3}{2}\rangle - 0.37|\frac{7}{2}\rangle$
5	$-0.75i|-\frac{7}{2}\rangle + 0.13|-\frac{3}{2}\rangle - 0.32i|\frac{1}{2}\rangle + 0.55|\frac{5}{2}\rangle$
6	$-0.56i|-\frac{5}{2}\rangle + 0.44|-\frac{1}{2}\rangle + 0.70|\frac{7}{2}\rangle$
7	$-0.46i|-\frac{7}{2}\rangle + 0.53|-\frac{3}{2}\rangle + 0.54i|\frac{1}{2}\rangle - 0.44i|\frac{5}{2}\rangle$
8	$0.33i|-\frac{5}{2}\rangle - 0.48|-\frac{1}{2}\rangle - 0.55i|\frac{3}{2}\rangle + 0.60|\frac{7}{2}\rangle$

TABLE S4. Rabi frequencies arising from oscillations between the nuclear levels shown in Table S1 for the electronic configuration $[4f^5 5d^1]6s^2$ (CAS6-12) at the bridge site when a magnetic field of 0.5 T is applied along the z axis. A time-dependent electric field of 0.002 a.u. is applied along the z axis.

	Rabi frequency (MHz)
$1 \leftrightarrow 3$	0.07
$2 \leftrightarrow 4$	0.06
$3 \leftrightarrow 5$	0.23
$4 \leftrightarrow 6$	0.04
$5 \leftrightarrow 7$	0.36
$6 \leftrightarrow 8$	0.34
$1 \leftrightarrow 5$	0.25
$2 \leftrightarrow 6$	0.25
$3 \leftrightarrow 7$	0.34
$4 \leftrightarrow 8$	0.35
$1 \leftrightarrow 7$	0.07
$2 \leftrightarrow 8$	0.04

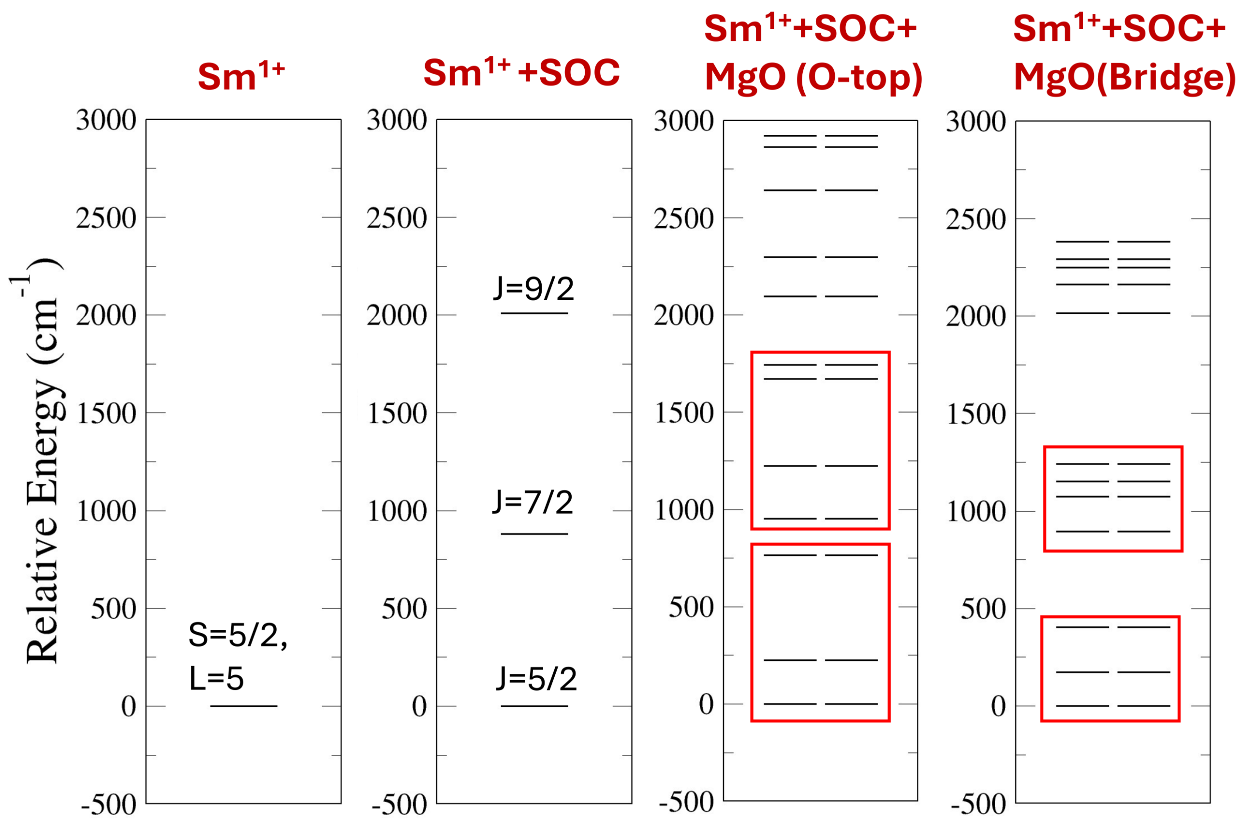

FIG. S3. Electronic structures of an isolated Sm^{1+} ion and a Sm^{1+} adatom on MgO for the electronic configuration $[4f^5]5d^06s^2$ (CAS5-7) at the O-top site and bridge site.

TABLE S5. Low-lying electronic energies of the Sm^{1+} case for the electronic configuration $[4f^5]5d^06s^2$ (CAS5-7) at the O-top and bridge sites as well as for an isolated Sm^{1+} atom

isolated atom		O-top	Bridge
Energy (cm^{-1})	J	Energy (cm^{-1})	Energy (cm^{-1})
0	$\frac{5}{2}$	0	0
0	$\frac{5}{2}$	0	0
0	$\frac{5}{2}$	225.3	173.6
0	$\frac{5}{2}$	225.3	173.6
0	$\frac{5}{2}$	764.0	402.9
0	$\frac{5}{2}$	764.0	402.9
879.5	$\frac{7}{2}$	953.0	896.3
879.5	$\frac{7}{2}$	953.0	896.3
879.5	$\frac{7}{2}$	1224.4	1074.9
879.5	$\frac{7}{2}$	1224.4	1074.9
879.5	$\frac{7}{2}$	1670.4	1152.2
879.5	$\frac{7}{2}$	1670.4	1152.2
879.5	$\frac{7}{2}$	1743.3	1242.2
879.5	$\frac{7}{2}$	1743.3	1242.2

TABLE S6. Calculated crystal-field parameters (in cm^{-1}) of the first-excited electronic multiplet $J = 5/2$ in the Sm^{1+} case for the electronic configuration $[4f^5]5d^06s^2$ (CAS5-7) at the bridge site.

B_k^q	Bridge
B_2^0	20.1644
B_2^2	-5.0457
B_4^0	-0.0893
B_4^2	1.6853
B_4^4	-1.7463

TABLE S7. Rabi frequencies arising from oscillations between the electronic-nuclear levels for the electronic configuration $[4f^5]5d^0 6s^2$ (CAS5-7) at the O-top site when a magnetic field of 2.0 T is applied along the z axis. A time-dependent electric field whose amplitude 0.002 a.u. is applied along the x axis.

	Rabi frequency (MHz)		Rabi frequency (MHz)
$\lvert -\frac{7}{2}, -\frac{1}{2}\rangle \leftrightarrow \lvert -\frac{5}{2}, -\frac{1}{2}\rangle$	0.67	$\lvert -\frac{7}{2}, \frac{1}{2}\rangle \leftrightarrow \lvert -\frac{5}{2}, \frac{1}{2}\rangle$	0.18
$\lvert -\frac{5}{2}, -\frac{1}{2}\rangle \leftrightarrow \lvert -\frac{3}{2}, -\frac{1}{2}\rangle$	0.76	$\lvert -\frac{5}{2}, \frac{1}{2}\rangle \leftrightarrow \lvert -\frac{3}{2}, \frac{1}{2}\rangle$	0.32
$\lvert -\frac{3}{2}, -\frac{1}{2}\rangle \leftrightarrow \lvert -\frac{1}{2}, -\frac{1}{2}\rangle$	0.71	$\lvert -\frac{3}{2}, \frac{1}{2}\rangle \leftrightarrow \lvert -\frac{1}{2}, \frac{1}{2}\rangle$	0.46
$\lvert -\frac{1}{2}, -\frac{1}{2}\rangle \leftrightarrow \lvert \frac{1}{2}, -\frac{1}{2}\rangle$	0.60	$\lvert -\frac{1}{2}, \frac{1}{2}\rangle \leftrightarrow \lvert \frac{1}{2}, \frac{1}{2}\rangle$	0.58
$\lvert \frac{1}{2}, -\frac{1}{2}\rangle \leftrightarrow \lvert \frac{3}{2}, -\frac{1}{2}\rangle$	0.44	$\lvert \frac{1}{2}, \frac{1}{2}\rangle \leftrightarrow \lvert \frac{3}{2}, \frac{1}{2}\rangle$	0.65
$\lvert \frac{3}{2}, -\frac{1}{2}\rangle \leftrightarrow \lvert \frac{5}{2}, -\frac{1}{2}\rangle$	0.27	$\lvert \frac{3}{2}, \frac{1}{2}\rangle \leftrightarrow \lvert \frac{5}{2}, \frac{1}{2}\rangle$	0.67
$\lvert \frac{5}{2}, -\frac{1}{2}\rangle \leftrightarrow \lvert \frac{7}{2}, -\frac{1}{2}\rangle$	0.12	$\lvert \frac{5}{2}, \frac{1}{2}\rangle \leftrightarrow \lvert \frac{7}{2}, \frac{1}{2}\rangle$	0.58
$\lvert -\frac{7}{2}, -\frac{1}{2}\rangle \leftrightarrow \lvert -\frac{3}{2}, -\frac{1}{2}\rangle$	0.25	$\lvert -\frac{7}{2}, \frac{1}{2}\rangle \leftrightarrow \lvert -\frac{3}{2}, \frac{1}{2}\rangle$	0.29
$\lvert -\frac{5}{2}, -\frac{1}{2}\rangle \leftrightarrow \lvert -\frac{1}{2}, -\frac{1}{2}\rangle$	0.36	$\lvert -\frac{5}{2}, \frac{1}{2}\rangle \leftrightarrow \lvert -\frac{1}{2}, \frac{1}{2}\rangle$	0.43
$\lvert -\frac{3}{2}, -\frac{1}{2}\rangle \leftrightarrow \lvert \frac{1}{2}, -\frac{1}{2}\rangle$	0.42	$\lvert -\frac{3}{2}, \frac{1}{2}\rangle \leftrightarrow \lvert \frac{1}{2}, \frac{1}{2}\rangle$	0.49
$\lvert -\frac{1}{2}, -\frac{1}{2}\rangle \leftrightarrow \lvert \frac{3}{2}, -\frac{1}{2}\rangle$	0.43	$\lvert -\frac{1}{2}, \frac{1}{2}\rangle \leftrightarrow \lvert \frac{3}{2}, \frac{1}{2}\rangle$	0.50
$\lvert \frac{1}{2}, -\frac{1}{2}\rangle \leftrightarrow \lvert \frac{5}{2}, -\frac{1}{2}\rangle$	0.37	$\lvert \frac{1}{2}, \frac{1}{2}\rangle \leftrightarrow \lvert \frac{5}{2}, \frac{1}{2}\rangle$	0.44
$\lvert \frac{3}{2}, -\frac{1}{2}\rangle \leftrightarrow \lvert \frac{7}{2}, -\frac{1}{2}\rangle$	0.26	$\lvert \frac{3}{2}, \frac{1}{2}\rangle \leftrightarrow \lvert \frac{7}{2}, \frac{1}{2}\rangle$	0.30
$\lvert -\frac{7}{2}, -\frac{1}{2}\rangle \leftrightarrow \lvert -\frac{7}{2}, \frac{1}{2}\rangle$	1.00	$\lvert \frac{7}{2}, -\frac{1}{2}\rangle \leftrightarrow \lvert \frac{7}{2}, \frac{1}{2}\rangle$	1.06
$\lvert -\frac{5}{2}, -\frac{1}{2}\rangle \leftrightarrow \lvert -\frac{5}{2}, \frac{1}{2}\rangle$	0.69	$\lvert \frac{5}{2}, -\frac{1}{2}\rangle \leftrightarrow \lvert \frac{5}{2}, \frac{1}{2}\rangle$	0.78
$\lvert -\frac{3}{2}, -\frac{1}{2}\rangle \leftrightarrow \lvert -\frac{3}{2}, \frac{1}{2}\rangle$	0.38	$\lvert \frac{3}{2}, -\frac{1}{2}\rangle \leftrightarrow \lvert \frac{3}{2}, \frac{1}{2}\rangle$	0.50
$\lvert -\frac{1}{2}, -\frac{1}{2}\rangle \leftrightarrow \lvert -\frac{1}{2}, \frac{1}{2}\rangle$	0.08	$\lvert \frac{1}{2}, -\frac{1}{2}\rangle \leftrightarrow \lvert \frac{1}{2}, \frac{1}{2}\rangle$	0.22

TABLE S8. Eight low-lying electronic-nuclear eigenstates for the electronic ground state of the electronic configuration $[4f^5]5d^06s^2$ (CAS5-7) at the bridge site when a magnetic field of 2.0 T is applied along the z axis. Only coefficients greater than 0.1 are shown.

State #	Eigenstate
1	$0.28i\|-\frac{7}{2},-\frac{1}{2}\rangle + 0.75\|-\frac{3}{2},-\frac{1}{2}\rangle - 0.13i\|-\frac{1}{2},\frac{1}{2}\rangle - 0.56i\|\frac{1}{2},-\frac{1}{2}\rangle - 0.14\|\frac{5}{2},-\frac{1}{2}\rangle$
2	$0.57i\|-\frac{5}{2},-\frac{1}{2}\rangle + 0.13\|-\frac{3}{2},\frac{1}{2}\rangle + 0.73\|-\frac{1}{2},-\frac{1}{2}\rangle - 0.10i\|\frac{1}{2},\frac{1}{2}\rangle - 0.33i\|\frac{3}{2},-\frac{1}{2}\rangle$
3	$0.78\|-\frac{7}{2},-\frac{1}{2}\rangle - 0.15i\|-\frac{3}{2},-\frac{1}{2}\rangle + 0.53\|\frac{1}{2},-\frac{1}{2}\rangle - 0.24i\|\frac{5}{2},-\frac{1}{2}\rangle$
4	$0.77\|-\frac{5}{2},-\frac{1}{2}\rangle + 0.35i\|-\frac{1}{2},-\frac{1}{2}\rangle + 0.52\|\frac{3}{2},-\frac{1}{2}\rangle$
5	$-0.54i\|-\frac{7}{2},-\frac{1}{2}\rangle + 0.61\|-\frac{3}{2},-\frac{1}{2}\rangle + 0.45i\|\frac{1}{2},-\frac{1}{2}\rangle + 0.35\|\frac{5}{2},-\frac{1}{2}\rangle$
6	$-0.28\|-\frac{5}{2},-\frac{1}{2}\rangle - 0.56i\|-\frac{1}{2},-\frac{1}{2}\rangle + 0.73\|\frac{3}{2},-\frac{1}{2}\rangle - 0.24i\|\frac{7}{2},-\frac{1}{2}\rangle$
7	$-0.16\|-\frac{3}{2},-\frac{1}{2}\rangle - 0.43i\|\frac{1}{2},-\frac{1}{2}\rangle + 0.88\|\frac{5}{2},-\frac{1}{2}\rangle$
8	$-0.26i\|\frac{3}{2},-\frac{1}{2}\rangle + 0.96\|\frac{7}{2},-\frac{1}{2}\rangle$

TABLE S9. Rabi frequencies arising from oscillations between the electronic-nuclear levels shown in Table S7 for the electronic configuration $[4f^5]5d^06s^2$ (CAS5-7) at the bridge site when a magnetic field of 2.0 T is applied along the z axis. A time-dependent electric field of 0.002 a.u. is applied along the z axis.

	Rabi frequency (MHz)
$1 \leftrightarrow 3$	14.38
$2 \leftrightarrow 4$	15.26
$3 \leftrightarrow 5$	20.26
$4 \leftrightarrow 6$	17.71
$5 \leftrightarrow 7$	13.88
$6 \leftrightarrow 8$	9.72
$1 \leftrightarrow 5$	6.35
$2 \leftrightarrow 6$	3.92
$3 \leftrightarrow 7$	5.98
$4 \leftrightarrow 8$	1.82
$1 \leftrightarrow 7$	1.45
$2 \leftrightarrow 8$	0.33

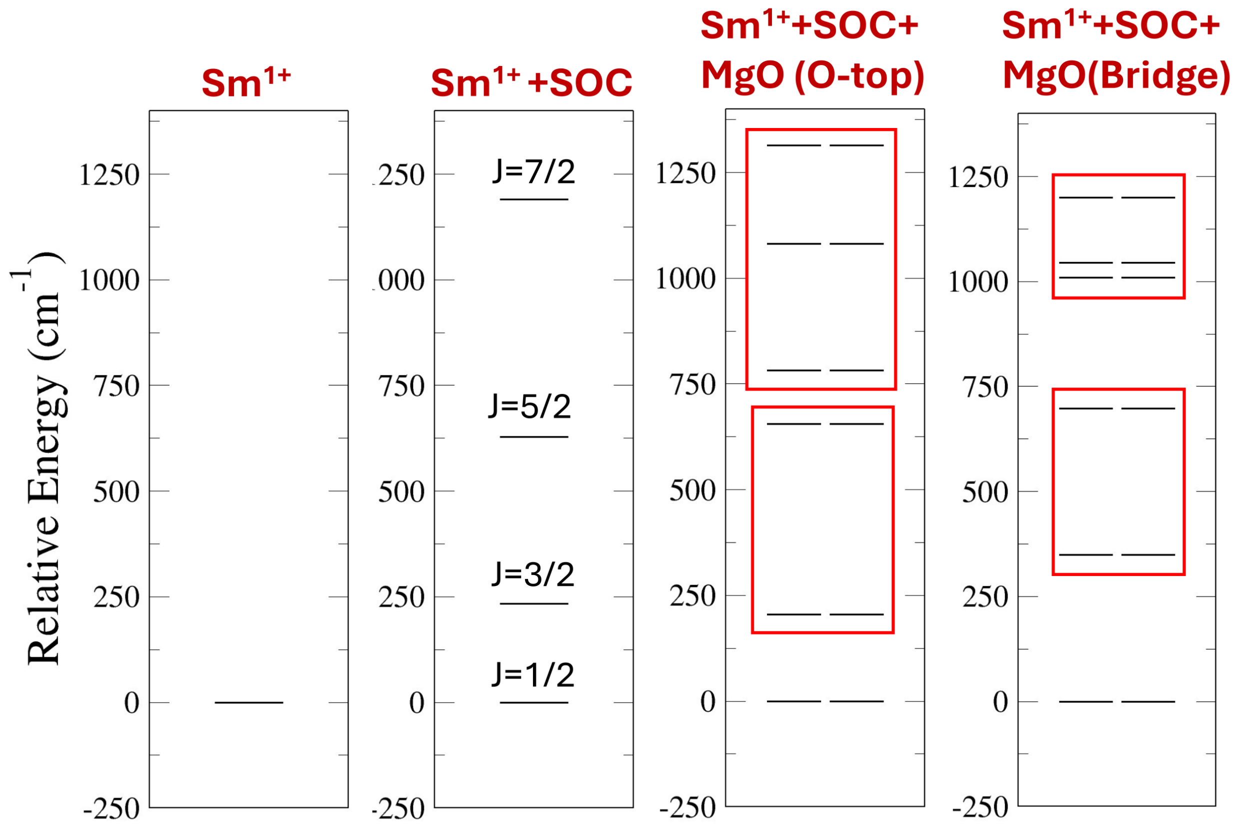

FIG. S4. Electronic structures of a neutral isolated Sm atom and a neutral Sm adatom on MgO for the electronic configuration $[4f^6 6s^1]5d^0$ (CAS7-8) at the O-top and bridge sites. Since spin-orbit coupling is comparable to the crystal field, effective J values cannot be assigned to excited electronic multiplets.

TABLE S10. Low-lying electronic energies of the Sm^{1+} case for the electronic configuration $[4f^6 6s^1]5d^0$ (CAS7-8) at the O-top and bridge sites as well as for an isolated Sm^{1+} atom

isolated atom		O-top	Bridge
Energy (cm^{-1})	J	Energy (cm^{-1})	Energy (cm^{-1})
0	$\frac{1}{2}$	0	0
0	$\frac{1}{2}$	0	0
233.7	$\frac{3}{2}$	204.7	348.7
233.7	$\frac{3}{2}$	204.7	348.7
233.7	$\frac{3}{2}$	655.6	697.6
233.7	$\frac{3}{2}$	655.6	697.6
628.5	$\frac{5}{2}$	781.4	1008.6
628.5	$\frac{5}{2}$	781.4	1008.6
628.5	$\frac{5}{2}$	1080.7	1045.2
628.5	$\frac{5}{2}$	1080.7	1045.2
628.5	$\frac{5}{2}$	1314.2	1199.8
628.5	$\frac{5}{2}$	1314.2	1199.8

TABLE S11. Additional Rabi frequencies arising from oscillations between the electronic-nuclear levels for the electronic configuration $[4f^6 6s^1]5d^0$ (CAS7-8) at the O-top site when a magnetic field of 2.0 T is applied along the z axis. A time-dependent electric field whose amplitude 0.002 a.u. is applied along the x axis.

	Rabi frequency (MHz)		Rabi frequency (MHz)
$\lvert -\frac{7}{2}, -\frac{1}{2}\rangle \leftrightarrow \lvert -\frac{7}{2}, \frac{1}{2}\rangle$	32.33	$\lvert \frac{7}{2}, -\frac{1}{2}\rangle \leftrightarrow \lvert \frac{7}{2}, \frac{1}{2}\rangle$	33.30
$\lvert -\frac{5}{2}, -\frac{1}{2}\rangle \leftrightarrow \lvert -\frac{5}{2}, \frac{1}{2}\rangle$	22.03	$\lvert \frac{5}{2}, -\frac{1}{2}\rangle \leftrightarrow \lvert \frac{5}{2}, \frac{1}{2}\rangle$	24.82
$\lvert -\frac{3}{2}, -\frac{1}{2}\rangle \leftrightarrow \lvert -\frac{3}{2}, \frac{1}{2}\rangle$	12.06	$\lvert \frac{3}{2}, -\frac{1}{2}\rangle \leftrightarrow \lvert \frac{3}{2}, \frac{1}{2}\rangle$	16.04
$\lvert -\frac{1}{2}, -\frac{1}{2}\rangle \leftrightarrow \lvert -\frac{1}{2}, \frac{1}{2}\rangle$	2.39	$\lvert \frac{1}{2}, -\frac{1}{2}\rangle \leftrightarrow \lvert \frac{1}{2}, \frac{1}{2}\rangle$	6.97

TABLE S12. Eight low-lying electronic-nuclear eigenstates for the electronic ground state of the electronic configuration $[4f^6 6s^1]5d^0$ (CAS7-8) at the bridge site when a magnetic field of 2.0 T is applied along the z axis. Only coefficients greater than 0.1 are shown.

State #	Eigenstate
1	$0.22i\|-\frac{7}{2},-\frac{1}{2}\rangle + 0.69\|-\frac{3}{2},-\frac{1}{2}\rangle - 0.16i\|-\frac{1}{2},\frac{1}{2}\rangle - 0.62i\|\frac{1}{2},-\frac{1}{2}\rangle - 0.20\|\frac{5}{2},-\frac{1}{2}\rangle$
2	$0.48i\|-\frac{5}{2},-\frac{1}{2}\rangle + 0.14\|-\frac{3}{2},\frac{1}{2}\rangle + 0.75\|-\frac{1}{2},-\frac{1}{2}\rangle - 0.13i\|\frac{1}{2},\frac{1}{2}\rangle - 0.40i\|\frac{3}{2},-\frac{1}{2}\rangle$
3	$0.63\|-\frac{7}{2},-\frac{1}{2}\rangle - 0.40i\|-\frac{3}{2},-\frac{1}{2}\rangle + 0.54\|\frac{1}{2},-\frac{1}{2}\rangle - 0.10i\|\frac{3}{2},\frac{1}{2}\rangle - 0.37i\|\frac{5}{2},-\frac{1}{2}\rangle$
4	$0.76\|-\frac{5}{2},-\frac{1}{2}\rangle + 0.15i\|-\frac{1}{2},-\frac{1}{2}\rangle + 0.59\|\frac{3}{2},-\frac{1}{2}\rangle - 0.15\|\frac{7}{2},-\frac{1}{2}\rangle$
5	$0.73i\|-\frac{7}{2},-\frac{1}{2}\rangle + 0.52i\|-\frac{3}{2},-\frac{1}{2}\rangle - 0.20\|\frac{1}{2},-\frac{1}{2}\rangle + 0.38i\|\frac{5}{2},-\frac{1}{2}\rangle$
6	$-0.41\mathrm{i}\|-\frac{5}{2},-\frac{1}{2}\rangle + 0.61\|-\frac{1}{2},-\frac{1}{2}\rangle + 0.60i\|\frac{3}{2},-\frac{1}{2}\rangle + 0.30\|\frac{7}{2},-\frac{1}{2}\rangle$
7	$0.11i\|-\frac{7}{2},-\frac{1}{2}\rangle - 0.26\|-\frac{3}{2},-\frac{1}{2}\rangle - 0.50i\|\frac{1}{2},-\frac{1}{2}\rangle + 0.81\|\frac{5}{2},-\frac{1}{2}\rangle$
8	$-0.12\|-\frac{1}{2},-\frac{1}{2}\rangle - 0.32i\|\frac{3}{2},-\frac{1}{2}\rangle + 0.94\|\frac{7}{2},-\frac{1}{2}\rangle$

TABLE S13. Rabi frequencies arising from oscillations between the electronic-nuclear levels shown in Table S7 for the electronic configuration $[4f^6 6s^1]5d^0$ (CAS7-8) at the bridge site when a magnetic field of 2.0 T is applied along the z axis. A time-dependent electric field of 0.002 a.u. is applied along the z axis.

	Rabi frequency (MHz)
$1 \leftrightarrow 3$	9.20
$2 \leftrightarrow 4$	9.23
$3 \leftrightarrow 5$	13.38
$4 \leftrightarrow 6$	10.94
$5 \leftrightarrow 7$	8.50
$6 \leftrightarrow 8$	4.68
$1 \leftrightarrow 5$	1.14
$2 \leftrightarrow 6$	0.62
$3 \leftrightarrow 7$	3.10
$4 \leftrightarrow 8$	0.46
$1 \leftrightarrow 7$	0.07
$2 \leftrightarrow 8$	0.02

TABLE S14. Low-lying electronic energies of the neutral Sm case for the electronic configuration $[4f^5 5d^1 6s^1]$ (CAS7-13) at the O-top and bridge sites

O-top Energy (cm^{-1})	Bridge Energy (cm^{-1})
0	0
0	0
145.6	191.5
145.6	191.5
557.4	487.4
557.4	487.4
626.2	629.7
626.2	629.7
811.5	796.6
811.5	796.6
1196.5	1191.2
1196.5	1191.2

TABLE S15. Additional Rabi frequencies arising from oscillations between the electronic-nuclear levels for the electronic configuration $[4f^5 6s^1 5d^1]$ (CAS7-13) at the O-top site when a magnetic field of 2.0 T is applied along the z axis. A time-dependent electric field whose amplitude 0.002 a.u. is applied along the x axis.

	Rabi frequency (MHz)		Rabi frequency (MHz)
$\lvert -\frac{7}{2},-\frac{1}{2}\rangle \leftrightarrow \lvert -\frac{3}{2},-\frac{1}{2}\rangle$	0.08	$\lvert -\frac{7}{2},\frac{1}{2}\rangle \leftrightarrow \lvert -\frac{3}{2},\frac{1}{2}\rangle$	0.33
$\lvert -\frac{5}{2},-\frac{1}{2}\rangle \leftrightarrow \lvert -\frac{1}{2},-\frac{1}{2}\rangle$	0.17	$\lvert -\frac{5}{2},\frac{1}{2}\rangle \leftrightarrow \lvert -\frac{1}{2},\frac{1}{2}\rangle$	0.52
$\lvert -\frac{3}{2},-\frac{1}{2}\rangle \leftrightarrow \lvert \frac{1}{2},-\frac{1}{2}\rangle$	0.26	$\lvert -\frac{3}{2},\frac{1}{2}\rangle \leftrightarrow \lvert \frac{1}{2},\frac{1}{2}\rangle$	0.66
$\lvert -\frac{1}{2},-\frac{1}{2}\rangle \leftrightarrow \lvert \frac{3}{2},-\frac{1}{2}\rangle$	0.36	$\lvert -\frac{1}{2},\frac{1}{2}\rangle \leftrightarrow \lvert \frac{3}{2},\frac{1}{2}\rangle$	0.73
$\lvert \frac{1}{2},-\frac{1}{2}\rangle \leftrightarrow \lvert \frac{5}{2},-\frac{1}{2}\rangle$	0.45	$\lvert \frac{1}{2},\frac{1}{2}\rangle \leftrightarrow \lvert \frac{5}{2},\frac{1}{2}\rangle$	0.72
$\lvert \frac{3}{2},-\frac{1}{2}\rangle \leftrightarrow \lvert \frac{7}{2},-\frac{1}{2}\rangle$	0.45	$\lvert \frac{3}{2},\frac{1}{2}\rangle \leftrightarrow \lvert \frac{7}{2},\frac{1}{2}\rangle$	0.58

TABLE S16. Eight low-lying electronic-nuclear eigenstates for the electronic ground state of the electronic configuration $[4f^5 6s^1 5d^1]$ (CAS7-13) at the bridge site when a magnetic field of 2.0 T is applied along the z axis. Only coefficients greater than 0.1 are shown.

State #	Eigenstate
1	$-0.11\vert-\frac{7}{2},-\frac{1}{2}\rangle - 0.22i\vert-\frac{5}{2},\frac{1}{2}\rangle - 0.48i\vert-\frac{3}{2},-\frac{1}{2}\rangle + 0.46\vert-\frac{1}{2},\frac{1}{2}\rangle + 0.57\vert\frac{1}{2},-\frac{1}{2}\rangle + 0.34i\vert\frac{3}{2},\frac{1}{2}\rangle$ $+0.23i\vert\frac{5}{2},-\frac{1}{2}\rangle$
2	$-0.29i\vert-\frac{5}{2},-\frac{1}{2}\rangle + 0.37\vert-\frac{3}{2},\frac{1}{2}\rangle + 0.60\vert-\frac{1}{2},-\frac{1}{2}\rangle + 0.45i\vert\frac{1}{2},\frac{1}{2}\rangle + 0.42i\vert\frac{3}{2},-\frac{1}{2}\rangle - 0.19\vert\frac{5}{2},\frac{1}{2}\rangle$
3	$0.20\vert-\frac{7}{2},\frac{1}{2}\rangle + 0.56\vert-\frac{5}{2},-\frac{1}{2}\rangle + 0.34i\vert-\frac{3}{2},\frac{1}{2}\rangle + 0.17i\vert-\frac{1}{2},-\frac{1}{2}\rangle + 0.23\vert\frac{1}{2},\frac{1}{2}\rangle + 0.52\vert\frac{3}{2},-\frac{1}{2}\rangle$ $+0.35i\vert\frac{5}{2},\frac{1}{2}\rangle + 0.22i\vert\frac{7}{2},-\frac{1}{2}\rangle$
4	$-0.32i\vert-\frac{7}{2},-\frac{1}{2}\rangle + 0.36\vert-\frac{5}{2},\frac{1}{2}\rangle + 0.53\vert-\frac{3}{2},-\frac{1}{2}\rangle - 0.28i\vert\frac{1}{2},-\frac{1}{2}\rangle + 0.40\vert\frac{3}{2},\frac{1}{2}\rangle + 0.46\vert\frac{5}{2},-\frac{1}{2}\rangle$ $+0.28\vert\frac{7}{2},\frac{1}{2}\rangle$
5	$0.64i\vert-\frac{7}{2},-\frac{1}{2}\rangle + 0.24i\vert-\frac{5}{2},\frac{1}{2}\rangle + 0.27\vert-\frac{1}{2},\frac{1}{2}\rangle + 0.32\vert\frac{1}{2},-\frac{1}{2}\rangle - 0.16i\vert\frac{3}{2},\frac{1}{2}\rangle - 0.51i\vert\frac{5}{2},-\frac{1}{2}\rangle$ $+0.27\vert\frac{7}{2},\frac{1}{2}\rangle$
6	$0.27\vert-\frac{7}{2},\frac{1}{2}\rangle + 0.58\vert-\frac{5}{2},-\frac{1}{2}\rangle - 0.48i\vert-\frac{1}{2},-\frac{1}{2}\rangle + 0.17\vert\frac{1}{2},\frac{1}{2}\rangle - 0.20\vert\frac{3}{2},-\frac{1}{2}\rangle - 0.38i\vert\frac{5}{2},\frac{1}{2}\rangle$ $-0.38i\vert\frac{7}{2},-\frac{1}{2}\rangle$
7	$0.62\vert-\frac{7}{2},-\frac{1}{2}\rangle - 0.48i\vert-\frac{3}{2},-\frac{1}{2}\rangle - 0.43\vert\frac{1}{2},-\frac{1}{2}\rangle + 0.36i\vert\frac{5}{2},-\frac{1}{2}\rangle - 0.26\vert\frac{7}{2},\frac{1}{2}\rangle$
8	$-0.14i\vert-\frac{7}{2},\frac{1}{2}\rangle - 0.21i\vert-\frac{5}{2},-\frac{1}{2}\rangle - 0.17\vert-\frac{3}{2},\frac{1}{2}\rangle - 0.31\vert-\frac{1}{2},-\frac{1}{2}\rangle + 0.22i\vert\frac{1}{2},\frac{1}{2}\rangle + 0.44i\vert\frac{3}{2},-\frac{1}{2}\rangle$ $+0.24\vert\frac{5}{2},\frac{1}{2}\rangle + 0.71\vert\frac{7}{2},-\frac{1}{2}\rangle$

TABLE S17. Rabi frequencies arising from oscillations between the electronic-nuclear levels shown in Table S10 for the electronic configuration [$4f^5 6s^1 5d^1$] (CAS7-13) at the bridge site when a magnetic field of 2.0 T is applied along the z axis. A time-dependent electric field of 0.002 a.u. is applied along the z axis.

	Rabi frequency (MHz)
$1 \leftrightarrow 4$	49.07
$2 \leftrightarrow 3$	49.07
$3 \leftrightarrow 6$	86.00
$4 \leftrightarrow 5$	85.91
$5 \leftrightarrow 7$	150.28
$6 \leftrightarrow 8$	114.56
$1 \leftrightarrow 5$	1.86
$2 \leftrightarrow 6$	1.90
$3 \leftrightarrow 8$	1.30
$4 \leftrightarrow 7$	6.46
$1 \leftrightarrow 7$	0.75
$2 \leftrightarrow 8$	0.66
